# Dose-Dependent Association Between Body Mass Index and Mental Health and Changes Over Time

**DOI:** 10.1001/jamapsychiatry.2024.0921

**Published:** 2024-05-15

**Authors:** Shanquan Chen, Hao Zhang, Min Gao, Daiane Borges Machado, Huajie Jin, Nathaniel Scherer, Wei Sun, Feng Sha, Tracey Smythe, Tamsin J. Ford, Hannah Kuper

**Affiliations:** 1 International Centre for Evidence in Disability, London School of Hygiene & Tropical Medicine, London, United Kingdom; 2 School of Public Health, Hangzhou Normal University, Hangzhou, China; 3 Nuffield Department of Primary Care Health, University of Oxford, Oxford, United Kingdom; 4 National Institute for Health and Care Research Oxford Health Biomedical Research Centre, Oxford University Hospitals National Health Service Foundation Trust, Oxford, United Kingdom; 5 Department of Global Health and Social Medicine, Harvard Medical School, Boston, Massachusetts; 6 Center of Data and Knowledge Integration for Health, Fiocruz, Salvador, Brazil; 7 Institute of Psychiatry, Psychology, and Neuroscience, King’s College London, London, United Kingdom; 8 Shenzhen Institute of Advanced Technology, Chinese Academy of Sciences, Shenzhen, Guangdong, China; 9 Division of Physiotherapy, Department of Health and Rehabilitation Sciences, Stellenbosch University, Cape Town, South Africa; 10 Department of Psychiatry, University of Cambridge, Cambridge, United Kingdom; 11 Cambridgeshire and Peterborough National Health Service Foundation Trust, Cambridge, United Kingdom

## Abstract

**Importance:**

Overweight and obesity affect 340 million adolescents worldwide and constitute a risk factor for poor mental health. Understanding the association between body mass index (BMI) and mental health in adolescents may help to address rising mental health issues; however, existing studies lack comprehensive evaluations spanning diverse countries and periods.

**Objective:**

To estimate the association between BMI and mental health and examine changes over time from 2002 to 2018.

**Design, Setting, and Participants:**

This was a repeated multicountry cross-sectional study conducted between 2002 and 2018 and utilizing data from the Health Behaviour in School-aged Children (HBSC) survey in Europe and North America. The study population consisted of more than 1 million adolescents aged 11 to 15 years, with all surveyed children included in the analysis. Data were analyzed from October 2022 to March 2023.

**Main Outcomes and Measures:**

Mental health difficulties were measured by an 8-item scale for psychological concerns, scoring from 0 to 32, where a higher score reflects greater psychosomatic issues. BMI was calculated using weight divided by height squared and adjusted for age and sex. Data were fitted by multilevel generalized additive model. Confounders included sex, living with parents, sibling presence, academic pressure, the experience of being bullied, family affluence, screen time, and physical activity.

**Results:**

Our analysis of 1 036 869 adolescents surveyed from 2002 to 2018, with a mean (SD) age of 13.55 (1.64) years and comprising 527 585 girls (50.9%), revealed a consistent U-shaped association between BMI and mental health. After accounting for confounders, adolescents with low body mass and overweight or obesity had increased psychosomatic symptoms compared to those with healthy weight (unstandardized β, 0.14; 95% CI, 0.08 to 0.19; unstandardized β, 0.27; 95% CI, 0.24 to 0.30; and unstandardized β, 0.62; 95% CI, 0.56 to 0.67, respectively), while adolescents with underweight had fewer symptoms (unstandardized β, −0.18; 95% CI, −0.22 to −0.15). This association was observed across different years, sex, and grade, indicating a broad relevance to adolescent mental health. Compared to 2002, psychosomatic concerns increased significantly in 2006 (unstandardized β, 0.19; 95% CI, 0.11 to 0.26), 2010 (unstandardized β, 0.14; 95% CI, 0.07 to 0.22), 2014 (unstandardized β, 0.48; 95% CI, 0.40 to 0.56), and 2018 (unstandardized β, 0.82; 95% CI, 0.74 to 0.89). Girls reported significantly higher psychosomatic concerns than boys (unstandardized β, 2.27; 95% CI, 2.25 to 2.30). Compared to primary school, psychosomatic concerns rose significantly in middle school (unstandardized β, 1.15; 95% CI, 1.12 to 1.18) and in high school (unstandardized β, 2.12; 95% CI, 2.09 to 2.15).

**Conclusions and Relevance:**

Our study revealed a U-shaped association between adolescent BMI and mental health, which was consistent across sex and grades and became stronger over time. These insights emphasize the need for targeted interventions addressing body image and mental health, and call for further research into underlying mechanisms.

## Key Points

**Question:**

How does the association between body mass index (BMI) and mental health change over time among adolescents, and do such associations and vary by sex and school grade?

**Findings:**

In this survey study analyzing more than 1 million adolescents from Europe and North America, a consistent U-shaped association was found between BMI and mental health, which grew stronger over time, especially among adolescents with low body mass.

**Meaning:**

The increasing association between BMI and adolescent mental health over time underscores the urgency for tailored interventions targeting body image and well-being.

## Teaser

This survey study evaluates the association between body mass index and mental health and changes in associations over time from 2002 to 2018.

## Introduction

Adolescence is a vital developmental stage with significant physical, emotional, and social changes, alongside unique health challenges, like the rising prevalence of obesity. This increase is primarily attributed to a biosocioecological model.^1^ Contributors include high-calorie diets, increased sedentary behavior due to technology, reduced physical activity, economic barriers to healthy eating and exercise, genetic factors, and marketing of unhealthy foods.^1,2^ In 2016, 340 million children and adolescents had overweight or obese globally.^3^ Higher weight not only carries risks of physical diseases, like cardiovascular disease and type 2 diabetes, but also affects current and future mental health.^4,5,6^ This issue is increasingly critical as the growing rates of overweight and obesity in adolescence often extend into early adulthood.^7,8^

The literature shows complex associations between body mass index (BMI) and mental health.^9,10,11,12,13,14,15,16,17,18,19,20^ Studies in South Korea, eastern London, and the US observed a U-shaped BMI–mental health association.^10,12,13^ However, these studies often focus on single countries^14,15,16,17^ or short periods,^18,19,20^ limiting a comprehensive analysis across multiple countries and over time. Examining data from various countries and over extended periods allows for a better understanding of these trends in different cultural contexts and the influence of societal changes on the association between BMI and mental health.

Previous studies suggest that biopsychosocial factors, like sex and age, might modify the association between BMI and mental health. Sex-based differences may arise from varying pubertal experiences, societal expectations, and body image perceptions.^6,21,22,23,24,25^ The association between BMI and mental health could also vary across school grades, reflecting the growing challenges adolescents face as they age.^5,26^ While sex-based differences in this association are known, evidence examining these trends over time and across educational backgrounds is scarce. Analyzing subgroups by sex and school grades could reveal important patterns relevant to this demographic.

In this study, we aimed to investigate temporal shifts in the association between BMI and mental health among adolescents using a large, multicountry sample of adolescents from Europe and North America, spanning the years 2002 to 2018. We also conducted subgroup analyses by sex and grade levels to better understand the dose-dependent BMI–mental health association in this key demographic.

## Method

### Data Source and Participants

We used data from the Health Behavior in School-Aged Children (HBSC) study, a World Health Organization (WHO)–affiliated multinational research project assessing the health, well-being, and behaviors of adolescents in Europe and North America. The HBSC collects data every 4 years from representative samples of 11-, 13-, and 15-year-olds in participating countries. The HBSC used a complex sampling design, including stratification and primary sampling units, for representative data collection.^27^ Stratification divides the target population into subgroups by age, geographical location, or school type, ensuring coverage of at least 95% of the eligible target population within the sample frame.^27^ Primary sampling units are typically schools or classes, selected systematically or randomly from all eligible schools.^27^ The HBSC minimized social desirability bias by ensuring anonymity, using standardized, back-translated questionnaires, and supervising survey completion in classrooms.^27^ The questionnaire, designed to be neutral and using validated scales, underwent pilot testing and postsurvey data checks for bias. Spanning 35 to 46 countries each round with a standardized protocol, the HBSC had a response rate over 60% in most countries.^28^ More details about HBSC methods are documented elsewhere.^27^ This study analyzed the latest 5 rounds of HBSC data from 2002 to 2018, which are publicly accessible. Its use of secondary deidentified data exempts it from institutional review board oversight. Each HBSC survey received ethics approval and informed consent from participants and their parents in the respective countries. Data were analyzed from October 2022 to March 2023. The study followed the Strengthening the Reporting of Observational Studies in Epidemiology (STROBE) reporting guideline.

### Outcome and Measures

Mental health was measured using an 8-item instrument assessing psychosomatic concerns (feeling low, irritability, nervousness, sleep difficulties, dizziness, headache, stomachache, and backache). Adolescents reported the frequency of these concerns over the past 6 months on a 5-point scale (daily to rarely/never). Responses were reverse coded (0–4) and summed to a total score (0-32), with higher scores indicating more psychosomatic concerns. This instrument was validated through qualitative interviews with adolescents for relevance and comprehensibility, and a quantitative test-retest method yielding satisfactory intraclass correlation coefficients between 0.61 and 0.75.^29^

BMI was calculated as weight in kilograms divided by height in meters squared across participating countries. Considering adolescents’ developmental stage, this study used age- and sex-standardized BMI (BMI *z* score) according to WHO standards.^30^ For dose-dependent analysis, adolescents were categorized into distinct BMI *z* score groups: low body mass (BMI *z* score ≤−2), underweight (BMI *z* score −1.99 to −1), healthy weight (BMI *z* score −0.99 to 0.99), overweight (BMI *z* score 1 to 1.99), and obese (BMI *z* score ≥2).^31^

### Confounders

We considered several confounders that previous literature has identified as relevant to both BMI and mental health. These included sociodemographic variables, like age, sex, and family affluence of adolescents.^4,18^ Family affluence was measured using the validated Family Affluence Scale, a proxy for socioeconomic status, with scores ranging from 0 to 9, where higher scores indicate greater affluence.^32^

We also investigated factors such as screen time, physical activity, living with parents, sibling presence, self-rated academic pressure, and experience of being bullying, given the established associations of these variables with mental health.^4,18,33,34,35,36^ These confounders were chosen based on the hypothesis that they could independently influence both BMI and mental health, rather than being on the causal pathway. Screen time included television, gaming, and other electronic device usage, with 9 options from none to ≥7 hours daily, averaged using a 5:2 weekday-to-weekend ratio. Physical activity was assessed by the number of days with at least 60 minutes of moderate to vigorous activity in the past week.

### Statistical Analysis

For basic description, we reported continuous data as means and SDs and categorical data as numbers and percentages. We also described the data by BMI *z *score categories and tested the differences between BMI *z *score categories by analysis of variance test for continuous variables and χ^2^ test for categorical variables.

We explored the association between BMI *z *score and mental health by fitting the data by a multilevel generalized additive model (GAM), with psychosomatic concerns as the outcome and BMI *z *score as the exposure, controlling for survey year, sex, living with parents, sibling presence, academic pressure, the experience of being bullied, family affluence, screen time, and physical activity. Among them, BMI, family affluence, screen time, and physical activity were represented as smoothing splines. The confounders were included in the model in a progressive way. To account for the nested structure of the data, the models included random intercept and random slope for BMI *z *score at the level of classroom, school, and country.

We quantified the dose-dependent associations between BMI *z *score and mental health by repeating the previously mentioned multilevel GAM analysis with the categorical BMI *z *score as the exposure, adjusted for the same confounders listed above in a progressive way. To assess the effect modification of sex or school grade on above associations, we also fitted the data by incorporating the following interaction terms into the model: categorical zBMI × sex, categorical zBMI × school grade, and categorical zBMI × sex × school grade. We further explored the association between BMI z score and mental health stratified by sex and/or school grade if there was evidence of an interaction, assessed by likelihood ratio test. To assess the potential temporal shifts in these dose-dependent associations and the corresponding variations within specific subgroups, we sequentially incorporated the following interaction terms into the model: categorical zBMI × survey year, categorical zBMI × survey year × sex, categorical zBMI × survey year × school grade, and categorical zBMI × survey year × sex × school grade.

To assess the association between survey year or sex and mental health, we fitted multilevel linear models with psychosomatic concerns as the outcome and year or sex as the exposure, without controlling for confounders due to no expected influencing factors. For the association between school grade and mental health, we employed multilevel GAM models, considering psychosomatic concerns as the outcome and school grade as the exposure, adjusting for sex, living with parents, sibling presence, and smoothing splines of family affluence. Confounders were progressively included in the model. All models featured random intercepts and slopes for exposure at classroom, school, and country levels.

Survey weights were used to account for sampling design (including the unequal probability of selection, clustering, and stratification). The weight values were directly obtained from the HBSC datasets. Detailed calculation methods can be found elsewhere.^27^ Fitted models were evaluated using diagnostic tests to determine if the degrees of freedom for a smooth were suitable. This assessment was based on results from generalized cross-validation.^37^ Residual plots were also used to assess the models.

Missing values for the study variables ranged from 0.8% for age to 18.4% for BMI. As the models were fitted in a progressive way, we implemented 20 multiple imputations by chained equations, using all variables included in the models, to avoid biases due to missing data.

We conducted 2 analyses to check the robustness of our estimations. First, we repeated the analyses without missing values imputation. Second, we adopted a cross-validation approach by dividing the dataset into 5 folds, repeating our model to each set, and comparing the coefficients across different folds.

All analyses were completed using R version 3.6.0 (R Foundation). We report 2 -tailed *P* values and 95% CIs throughout. *P* < .05 was considered statistically significant.

## Results

From the 2002 to 2018 HBSC data, 1 036 869 adolescents with a mean (SD) age of 13.55 (1.64) years were studied, including 527 585 girls (50.9%) (Table 1). Among them, 346 247 (33.4%) were in primary school, 774 431 (74.7%) lived with both parents, 887 171 (85.6%) had siblings, 119 567 (11.5%) felt academic pressure, and 308 944 (29.8%) reported having experienced bullying. The mean (SD) family affluence score was 5.47 (2.09), mean (SD) daily screen time was 6.06 (3.71) hours, and mean (SD) weekly physical activity was 4.04 (2.1) days. The mean (SD psychosomatic concern score was 8.15 (6.65). BMI *z *score category–stratified basic information in Table 1 shows significant differences in terms of age, sex, school grade, living arrangements, sibling presence, academic pressure, experience being bullied, family affluence, screen time, physical activity, and psychosomatic concerns. The distribution of missing values by BMI *z* score categories are presented in eTable 1 in Supplement 1. Adolescents with healthy weight, overweight, or obesity had greater proportions of missing values on age, school grade, and living with parents.

**Table 1.** Description of Study Population

Variable	No. (%)						*P* value^a^
	All (N = 1 036 869)	Low body mass (n = 54 767)	Underweight (n = 145 192)	Healthy weight (n = 635 198)	Overweight (n = 154 153)	Obese (n = 47 559)	
Survey year							
2002	162 305 (15.7)	9168 (16.7)	24 823 (17.1)	100 769 (15.9)	21 450 (13.9)	6095 (12.8)	<.001
2006	205 938 (19.9)	10 431 (19.0)	29 131 (20.1)	128 644 (20.3)	29 251 (19.0)	8481 (17.8)	
2010	213 595 (20.6)	10 255 (18.7)	28 761 (19.8)	131 911 (20.8)	32 403 (21.0)	10 265 (21.6)	
2014	214 080 (20.6)	11 203 (20.5)	29 286 (20.2)	130 413 (20.5)	32 947 (21.4)	10 231 (21.5)	
2018	240 951 (23.2)	13 710 (25.0)	33 191 (22.9)	143 461 (22.6)	38 102 (24.7)	12 487 (26.3)	
Age, mean (SD), y	13.55 (1.64)	13.23 (1.57)	13.55 (1.63)	13.66 (1.64)	13.34 (1.63)	13.18 (1.63)	<.001
Female sex	527 585 (50.9)	28 988 (52.9)	85 211 (58.7)	334 353 (52.6)	62 999 (40.9)	16 034 (33.7)	<.001
School grade							
Grade 5 (primary school)	346 247 (33.4)	22 399 (40.9)	47 935 (33.0)	195 362 (30.8)	59 466 (38.6)	21 085 (44.3)	<.001
Grade 7 (middle school)	359 466 (34.7)	20 038 (36.6)	51 982 (35.8)	219 790 (34.6)	52 826 (34.3)	14 830 (31.2)	
Grade 9 (high school)	331 156 (31.9)	12 330 (22.5)	45 275 (31.2)	220 046 (34.6)	41 861 (27.2)	11 644 (24.5)	
Living with parents							
Both	774 431 (74.7)	41 036 (74.9)	110 191 (75.9)	476 638 (75.0)	112 979 (73.3)	33 587 (70.6)	<.001
One of parents	223 807 (21.6)	11 528 (21.0)	30 094 (20.7)	135 762 (21.4)	34 773 (22.6)	11 650 (24.5)	
Neither	38 631 (3.7)	2203 (4.0)	4907 (3.4)	22 798 (3.6)	6401 (4.2)	2322 (4.9)	
Sibling presence, yes	887 171 (85.6)	47 415 (86.6)	125 709 (86.6)	544 586 (85.7)	129 868 (84.2)	39 593 (83.3)	<.001
Academic pressure							
Not at all	214 934 (20.7)	12 635 (23.1)	30 561 (21.0)	129 386 (20.4)	32 454 (21.1)	9898 (20.8)	<.001
A little	457 057 (44.1)	23 719 (43.3)	65 215 (44.9)	281 162 (44.3)	66 841 (43.4)	20 120 (42.3)	
Some	245 311 (23.7)	11 981 (21.9)	33 665 (23.2)	152 164 (24.0)	36 327 (23.6)	11 174 (23.5)	
A lot	119 567 (11.5)	6432 (11.7)	15 751 (10.8)	72 486 (11.4)	18 531 (12.0)	6367 (13.4)	
Experienced bullying							
Never	727 925 (70.2)	36 959 (67.5)	103 399 (71.2)	454 547 (71.6)	103 588 (67.2)	29 432 (61.9)	<.001
Once or twice	190 011 (18.3)	10532 (19.2)	26 559 (18.3)	113 476 (17.9)	29 718 (19.3)	9726 (20.5)	
2-3 Times per mo	46 949 (4.5)	2791 (5.1)	6074 (4.2)	26 922 (4.2)	8136 (5.3)	3026 (6.4)	
Once/wk	30 220 (2.9)	1807 (3.3)	3908 (2.7)	17 235 (2.7)	5234 (3.4)	2036 (4.3)	
Several times/wk	41 764 (4.0)	2678 (4.9)	5252 (3.6)	23 018 (3.6)	7477 (4.9)	3339 (7.0)	
Family Affluence Scale score, mean (SD)	5.47 (2.09)	5.40 (2.19)	5.50 (2.13)	5.49 (2.08)	5.43 (2.04)	5.34 (2.04)	<.001
Screen time, mean (SD), h/d)	6.06 (3.71)	6.00 (3.85)	5.90 (3.67)	6.00 (3.66)	6.28 (3.78)	6.67 (4.00)	<.001
Physical activity, mean (SD), d/wk	4.04 (2.10)	4.09 (2.17)	4.04 (2.11)	4.08 (2.09)	3.96 (2.09)	3.76 (2.13)	<.001
Psychosomatic concerns, mean (SD)^b^	8.16 (6.56)	8.20 (6.73)	8.00 (6.44)	8.13 (6.50)	8.24 (6.65)	8.71 (7.04)	<.001

^a^ *P* values of between–body mass index *z *score categories were extracted from analysis of variance test for continuous variables and χ^2^ test for categorical variables.

^b^ Measured using an 8-item instrument assessing feeling low, irritability, nervousness, sleep difficulties, dizziness, headache, stomachache, and backache with frequency reported on a 5-point scale from daily to rarely/never.

**Figure 1.**
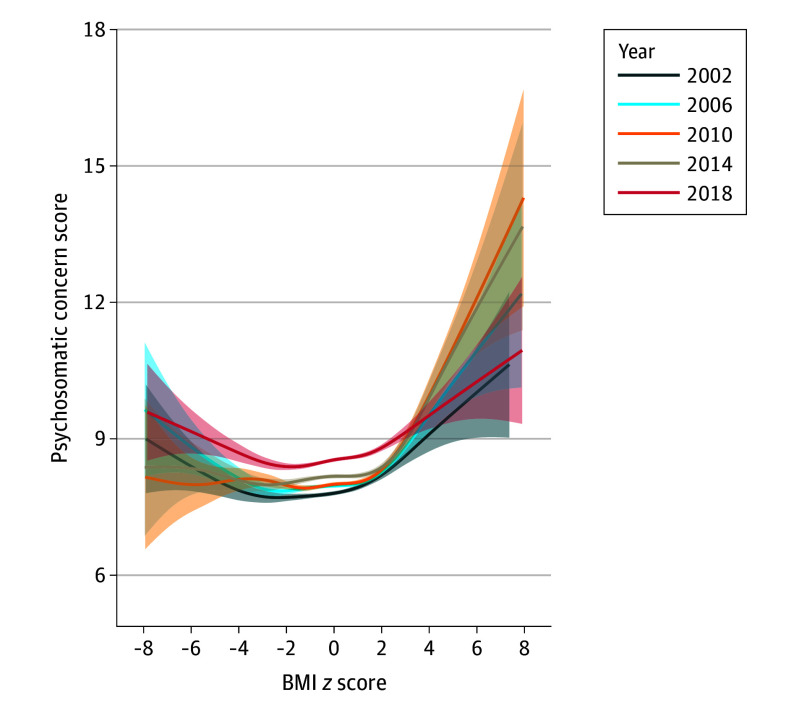
Generalized Additive Models of Psychosomatic Concerns as a Function of Body Mass Index (BMI) *z* Score by Survey Year The gray area presents 95% CIs of fitting on the function of BMI *z* score. The *P* value for spline of BMI *z *score was <.001. The *P* value was extracted from the multilevel generalized additive model, with psychosomatic concerns as the outcome and smooth term of BMI *z* score as the exposure, controlling for survey year, sex, grade, living with parents, sibling presence, academic pressure, having experienced bullying, and smooth term of survey year, Family Affluence Scale, screen time, and physical activity, as well as the random intercept and random slope for BMI *z* score at the level of classroom, school, and country. Psychosomatic concern scores were measured using an 8-item instrument assessing feeling low, irritability, nervousness, sleep difficulties, dizziness, headache, stomachache, and backache with frequency reported on a 5-point scale from daily to rarely/never.

Figure 1 reveals a significant nonlinear association between BMI *z *score and psychosomatic concerns, illustrating a U-shaped pattern. After controlling for confounders, compared to adolescents with healthy weight, those with low body mass had significantly higher psychosomatic symptoms(unstandardized β, 0.14; 95% CI, 0.08 to 0.19), those with underweight had significantly fewer psychosomatic symptoms (unstandardized β, −0.18; 95% CI, −0.22 to −0.15), and those with overweight or obesity had significantly higher psychosomatic symptoms (unstandardized β, 0.27; 95% CI, 0.24 to 0.30 and unstandardized β, 0.62; 95% CI, 0.56 to 0.67, respectively) (Table 2; eFigure 2A in Supplement 1). Compared to the survey conducted in 2002, the 2006 survey exhibited a significant increase in psychosomatic concerns (unstandardized β, 0.19; 95% CI, 0.11 to 0.26), as did the 2010 survey (unstandardized β, 0.14; 95% CI, 0.07 to 0.22), the 2014 survey (unstandardized β, 0.48; 95% CI, 0.40 to 0.56), and the 2018 survey (unstandardized β, 0.82; 95% CI, 0.74 to 0.89) (eTable 2 in Supplement 1). The U-shaped curve between BMI *z *score categories and psychosomatic concerns remained mostly constant over time, except for the steeper association among adolescents with low body mass (eFigure 2B in Supplement 1).

**Table 2.** Associations of Body Mass Index (BMI) *z* Score Categories With Psychosomatic Concerns

Variable	Confounders	BMI *z* score categories, unstandardized β (95% CI)^a^				
		Low body mass	Underweight	Healthy weight	Overweight	Obesity
Model 1.1	Univariable model	0.06 (0.01 to 0.12)	−0.13 (−0.17 to −0.10)	1 [Reference]	0.10 (0.06 to 0.13)	0.55 (0.49 to 0.61)
Model 1.2	Model 1.1 + survey year	0.06 (0.00 to 0.11)	−0.13 (−0.17 to −0.09)	1 [Reference]	0.08 (0.04 to 0.11)	0.52 (0.46 to 0.58)
Model 1.3	Model 1.2 + sex	0.05 (0.00 to 0.12)	−0.28 (−0.31 to −0.24)	1 [Reference]	0.36 (0.32 to 0.39)	0.97 (0.91 to 1.03)
Model 1.4	Model 1.3 + school grade	0.28 (0.22 to 0.33)	−0.21 (−0.25 to −0.18)	1 [Reference]	0.53 (0.49 to 0.56)	1.24 (1.19 to 1.30)
Model 1.5	Model 1.4 + living with parents	0.28 (0.22 to 0.33)	−0.21 (−0.24 to −0.17)	1 [Reference]	0.51 (0.47 to 0.54)	1.20 (1.14 to 1.25)
Model 1.6	model 1.5 + sibling presence	0.28 (0.22 to 0.33)	−0.21 (−0.24 to −0.17)	1 [Reference]	0.51 (0.47 to 0.54)	1.20 (1.14 to 1.26)
Model 1.7	Model 1.6 + family affluence	0.27 (0.22 to 0.33)	−0.20 (−0.24 to −0.17)	1 [Reference]	0.50 (0.47 to 0.54)	1.18 (1.12 to 1.24)
Model 1.8	Model 1.7 + screen time	0.27 (0.21 to 0.32)	−0.19 (−0.23 to −0.16)	1 [Reference]	0.46 (0.42 to 0.49)	1.07 (1.01 to 1.13)
Model 1.9	Model 1.8 + physical activity	0.26 (0.21 to 0.32)	−0.19 (−0.23 to −0.16)	1 [Reference]	0.42 (0.38 to 0.45)	0.99 (0.93 to 1.04)
Model 1.10	Model 1.9 + experience of been bullying	0.17 (0.11 to 0.22)	−0.19 (−0.22 to −0.15)	1 [Reference]	0.29 (0.26 to 0.32)	0.68 (0.62 to 0.73)
Model 1.11	Model 1.10 + academic pressure	0.14 (0.08 to 0.19)	−0.18 (−0.22 to −0.15)	1 [Reference]	0.27 (0.24 to 0.30)	0.62 (0.56 to 0.67)
						

^a^ Unstandardized βs and 95% CIs were extracted from multilevel generalized additive model, with psychosomatic concerns as the outcome and variable of interest as the exposure, as well as the random intercept and random slope for exposure at the level of classroom, school, and country.

**Figure 2.**
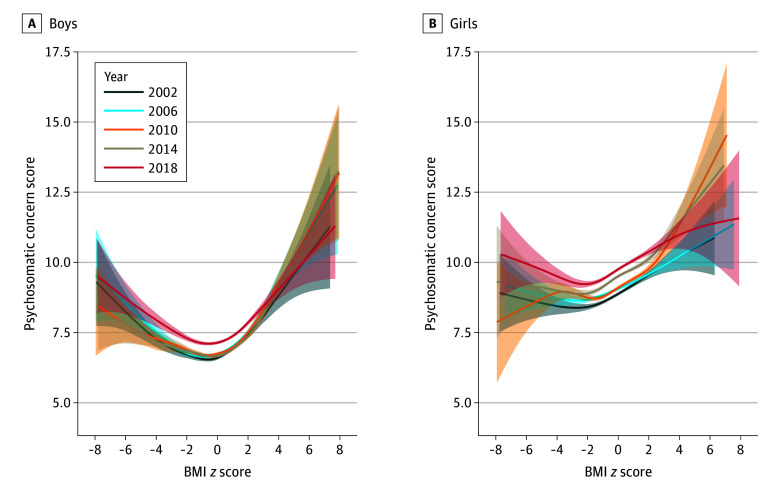
Generalized Additive Models of Psychosomatic Concerns as a Function of Body Mass Index (BMI) *z* score, by Survey Year and Sex The gray area presents 95% CIs of fitting on the function of BMI *z *score. For boys and girls, both *P* values for spline of BMI z score were <.001. *P* values were extracted from the multilevel generalized additive model, with psychosomatic concerns as the outcome and smooth term of BMI *z *score as the exposure, controlling for survey year, grade, living with parents, sibling presence, academic pressure, having experienced bullying, and smooth term of Family Affluence Scale, screen time, and physical activity, as well as the random intercept and random slope for BMI *z* score at the level of classroom, school, and country. Psychosomatic concern scores were measured using an 8-item instrument assessing feeling low, irritability, nervousness, sleep difficulties, dizziness, headache, stomachache, and backache with frequency reported on a 5-point scale from daily to rarely/never.

Figure 2 shows the results of the GAM evaluating psychosomatic concerns relative to BMI *z *score, stratified by sex. For the same BMI *z *score values, girls exhibited elevated levels of psychosomatic concerns compared to boys (unstandardized β, 2.27; 95% CI, 2.25 to 2.30) (eTable 2 in Supplement 1). The U-shaped association between BMI *z *score and psychosomatic concerns was more pronounced in boys (Figure 2; eFigure 2C in Supplement 1), and this sex disparity remained constant over time (eFigure 2E in Supplement 1).

**Figure 3.**
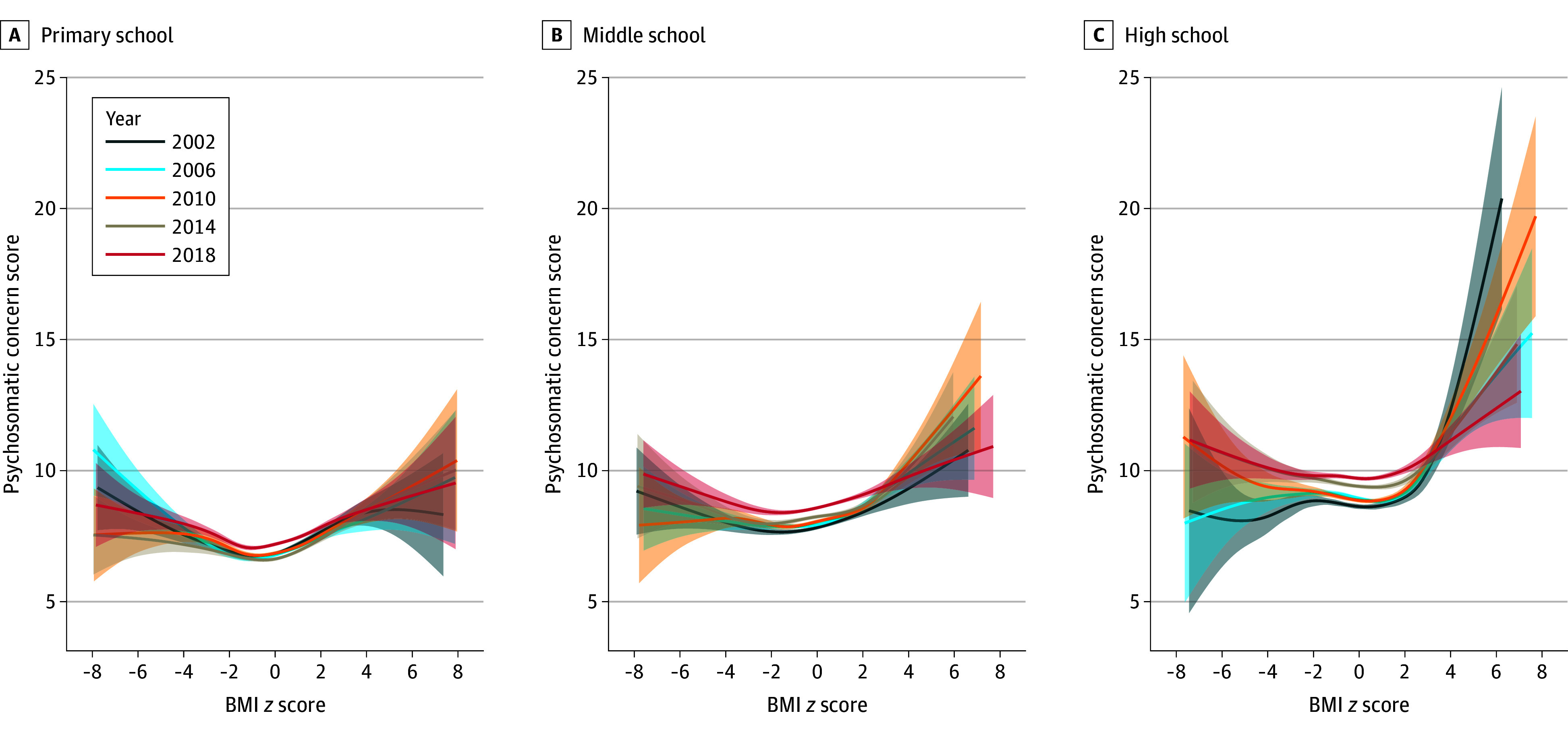
Generalized Additive Models of Psychosomatic Concerns as a Function of Body Mass Index (BMI) *z *score, by Survey Year and Grade The gray area presents 95% CIs of fitting on the function of BMI *z* score. For primary school, middle school, and high school, all *P* values for spline of BMI z score were <.001. *P* values were extracted from the multilevel generalized additive model, with psychosomatic concerns as the outcome and smooth term of BMI *z *score as the exposure, controlling for survey year, sex, living with parents, sibling presence, academic pressure, having experienced bullying, and smooth term of Family Affluence Scale, screen time, and physical activity, as well as the random intercept and random slope for BMI *z* score at the level of classroom, school, and country. Psychosomatic concern scores were measured using an 8-item instrument assessing feeling low, irritability, nervousness, sleep difficulties, dizziness, headache, stomachache, and backache with frequency reported on a 5-point scale from daily to rarely/never.

Figure 3 shows the results of the GAM evaluating psychosomatic concerns relative to BMI *z *score, stratified by school grade. Adolescents in middle school experienced a significant increase in psychosomatic concerns (unstandardized β, 1.17; 95% CI, 1.14 to 1.20) relative to those in primary school, while those in high school reported a significantly higher increase in psychosomatic concerns (unstandardized β, 2.18; 95% CI, 2.15 to 2.21) (eTable 2 in Supplement 1). The U-shaped curve became less pronounced with grade progression (Figure 3; eFigure 2D in Supplement 1), and this school grade disparity remained constant over time (eFigure 2F in Supplement 1).

eFigure 1 in Supplement 1 displays the results of the GAM evaluating psychosomatic concerns relative to BMI *z *score, stratified by sex and school grade. eFigure 1 in Supplement 1 reveals a greater disparity in school grades among girls with underweight and low body mass (eFigure 2G in Supplement 1), with consistent differences based on sex and school grade over time (eFigure 2H in Supplement 1).

The results from robustness checking without missing value imputation (eTable 3 and eFigures 3-7 in Supplement 1) and 5-fold cross-validation (eTables 4-5 in Supplement 1) confirm the consistency of the above main results.

## Discussion

This survey study used data from more than 1 million adolescents to assess the association between BMI and mental health among adolescents between 2002 and 2018. Overall, the association between BMI and mental health exhibited a U-shaped pattern, which was similar across survey year, sex, and grade. The lowest point located in the transitional range between underweight and healthy weight. Over time, the same BMI *z *score values were associated with increased mental health issues, a trend particularly pronounced in recent years. At the same BMI *z *score levels, girls exhibited a higher level of mental health issues than boys, and the U-shaped curve was steeper among boys. For the same BMI *z *score values, adolescents in higher grades experienced worse mental health. The U-shaped curve became less pronounced with grade progression, especially among girls. The identified disparities by sex or school grade remained constant over time.

Previous studies have shown a similar U-shaped association between BMI and mental health in specific adolescent groups^12,13^ and adults.^38,39^ This U-shaped association could be understood through a biosocioecological prospective. Individuals with underweight often experience a sense of insufficiency, negative body image, and societal or peer pressures, affecting mental health.^40^ Biological factors, like genetics, can make healthy weight maintenance challenging for some adolescents.^4^ Conversely, adolescents with overweight or obesity often experience stigmatization and bullying, contributing to mental health issues.^35,41,42,43^ The increase in obesity is often attributed to today’s obesogenic environment of unhealthy family and community lifestyles, aggressive marketing of unhealthy foods, promotion of sedentary behaviors, widespread availability of energy-dense foods, and increased screen time. This environment worsens obesity and the mental health challenges experienced by adolescents.^4,18^ Our findings, consistent across time, sex, and grades, confirm this U-shaped pattern, underscoring its relevance for public health policies. It is vital to address weight issues in adolescent mental health initiatives, focusing on challenges at both ends of the BMI *z *score spectrum.

The increasing mental health issues associated with the same BMI *z *score values over time, particularly in recent years, highlight a growing sensitivity among adolescents about body weight. The rise of social media and digital influence may underpin this development. This exposure to idealized body images may heighten self-consciousness about appearance and weight.^44,45^ Therefore, increased smartphone and other screen use and upward social comparisons prevalent in these environments may contribute to stronger associations between weight status and mental health issues in recent years.^46^ Additionally, societal stigma around having overweight or obesity could increase stress, anxiety, and depression in adolescents.^47,48,49,50^ During adolescence, when peer acceptance is crucial, deviation from societal norms can lead to alienation or low self-esteem.^51,52,53,54^ Despite a shift toward less discrimination against obesity due to a broader understanding of its causes,^1,55,56^ our study indicates that the association between weight and mental health in adolescents strengthened over time, highlighting the need for early interventions focusing on body image perceptions and mental health promotion.

Studies show a stronger association between body weight and mental health issues in girls than boys, with girls facing more severe body image and weight stigma.^6,57,58,59,60^ Our study confirms this in a broad, multinational adolescent group over time. We also found a dose-dependent association, with boys showing more sensitivity to BMI *z *score changes and girls with low body mass also increasingly affected, exacerbating mental health impacts. This new insight, not fully explored in previous research, deepens our understanding of the association between body weight and mental health in adolescent boys. It highlights the need for interventions targeting not only those at the extreme ends of the BMI *z *score spectrum but also those with minor BMI *z *score increases who might be at greater risk of mental health issues.

Adolescents in higher grades experienced worse mental health at the same BMI *z *score values, consistent with the increase in anxiety, depression, eating disorders, and self-harm with age.^9,34^ This decline may be due to heightened social sensitivity and social phobia as they progress through school.^9,34^ Additionally, the interplay of social, environmental, and pubertal changes affecting body shape could be influential. The transition from lower to higher school brings considerable changes in social dynamics, increased academic stress, and greater body consciousness, potentially intensifying weight-related mental health issues.^34,36,61,62^ Furthermore, as adolescents’ cognitive development evolves, their understanding of healthy body weight further impacts their mental health challenges.^63^

We found that the U-shaped curve between BMI *z* score and mental health flattened with grade progression, especially among girls in the underweight and low body mass range, suggesting they may have become less sensitive to body weight changes as they aged. This could result from increased body acceptance, maturity, and reduced sensitivity to weight-related stigma.^5,64^ Also, older adolescents’ self-esteem and self-perception may rely more on peer judgment and social feedback than actual body weight.^35,36^ Research shows adolescents seek body image validation^65^ and are heavily influenced by peers’ attitudes toward body image and mental health.^50,66^ Additionally, the surge in depression among adolescent girls might diminish the relative impact of BMI *z* scores, as their overall mental distress is already high. This could also explain the stronger BMI *z *score–body dissatisfaction association in girls, who often report greater body dissatisfaction.^4^ These findings highlight the need to consider various influences on adolescent mental health, including social and environmental factors, not just BMI *z *scores metrics.

### Strength and Limitations

This study’s strengths lie in its rigorous sampling and data collection methods, covering more than 1 million adolescents from various countries, providing a robust and statistically powerful sample. This large sample size marks an important improvement over previous studies. Spanning from 2002 to 2018, our study’s temporal scope exceeds that of prior research, offering a longitudinal view to identify trends in BMI *z *score and adolescent mental health, a feature often missed in earlier studies. We conducted a detailed analysis of BMI *z *score and mental health across sex and grade levels, uncovering patterns like increased susceptibility of boys to BMI *z *score changes and grade-related shifts in the association between BMI *z *score and mental health. These insights are crucial for creating more effective interventions. Our study also uniquely investigates grade progression’s effect mediating in this association, revealing a less pronounced U-shaped curve among older adolescents, particularly girls. Additionally, covering European and North American participants enhances the generalizability within these regions, although our focus on these areas without detailed country-level analysis suggests directions for future research.

This study also has limitations. First, using BMI *z* score as the only measure might miss aspects like muscle mass and fat distribution, potentially inaccurately classifying weight status in adolescents. Second, its cross-sectional design precludes establishing causal relationships between BMI *z* score and mental health. Third, mental health was measured by psychosomatic concerns, which do not cover all mental health manifestations in youth, and aspects like sleep duration and quality were not fully assessed. Fourth, the study does not consider confounders like dietary habits and family mental health history.^67,68,69,70^ Fifth, while demographically broad, it does not delve into race and ethnicity and cultural differences in body image perceptions. Future research should explore these cultural aspects and how they might influence the BMI–mental health association. Sixth, the data predate the COVID-19 pandemic, an event associated with increased mental health issues,^71^ leaving the impact of the pandemic on our findings uncertain. Future studies should investigate the pandemic’s effects on these associations.

## Conclusion

Our study of more than 1 million adolescents shows a persistent U-shaped association between BMI *z *score and mental health across different years, sex, and school grades. We noted a strengthening association between BMI *z *score and mental health over time, especially in boys and as students advance in school. These insights can inform public health and school programs, emphasizing correcting body image misconceptions, encouraging healthy weight, and creating supportive peer environments.

## Notes

Supplement 1. eTable 1. Distribution of missing values by zBMI categories. eTable 2. Associations of survey year, sex, and school grade, with psychosomatic concerns eTable 3. Associations of zBMI categories, survey year, sex, and school grade, with psychosomatic concerns, without imputation eTable 4. Associations of zBMI categories, survey year, sex, and school grade, with psychosomatic concerns, cross validation eFigure 1. Generalised additive models of psychosomatic concerns as a function of zBMI, by survey year by sex by grade eFigure 2. Joint associations and their temporal trends of zBMI and sub-group variables with psychosomatic concerns eFigure 3. Generalised additive models of psychosomatic concerns as a function of zBMI, by survey year, without imputation eFigure 4. Generalised additive models of psychosomatic concerns as a function of zBMI, by survey year by sex, without imputation eFigure 5. Generalised additive models of psychosomatic concerns as a function of zBMI, by survey year by grade, without imputation eFigure 6. Generalised additive models of psychosomatic concerns as a function of zBMI, by survey year by sex by grade, without imputation eFigure 7. Joint associations and their temporal trends of zBMI and sub-group variables with psychosomatic concerns, without imputation

Supplement 2. Data sharing statement
