# Supplementary material for: Dose-Dependent Association Between Body Mass Index and Mental Health and Changes Over Time
Source: JAMA Psychiatry. 2024 May 15;81(8):797–806. doi: 10.1001/jamapsychiatry.2024.0921 (PMC11097104; doi:10.1001/jamapsychiatry.2024.0921)
Supplement: Supplement 1. — eTable 1. Distribution of missing values by zBMI categories. eTable 2. Associations of survey year, sex, and school grade, with psychosomatic concerns eTable 3. Associations of zBMI categories, survey year, sex, and school grade, with psychosomatic concerns, without imputation eTable 4. Associations of zBMI categories, survey year, sex, and school grade, with psychosomatic concerns, cross validation eFigure 1. Generalised additive models of psychosomatic concerns as a function of zBMI, by survey year by sex by grade eFigure 2. Joint associations and their temporal trends of zBMI and sub-group variables with psychosomatic concerns eFigure 3. Generalised additive models of psychosomatic concerns as a function of zBMI, by survey year, without imputation eFigure 4. Generalised additive models of psychosomatic concerns as a function of zBMI, by survey year by sex, without imputation eFigure 5. Generalised additive models of psychosomatic concerns as a function of zBMI, by survey year by grade, without imputation eFigure 6. Generalised additive models of psychosomatic concerns as a function of zBMI, by survey year by sex by grade, without imputation eFigure 7. Joint associations and their temporal trends of zBMI and sub-group variables with psychosomatic concerns, without imputation [file jamapsychiatry-e240921-s001.pdf]

Supplemental Online Content

Chen S, Zhang H, Gao M, et al. Dose-dependent association between body mass index and mental health and changes over time. *JAMA Psychiatry*. Published online May 15, 2024. doi:10.1001/jamapsychiatry.2024.0921

eTable 1. Distribution of missing values by zBMI categories
eTable 2. Associations of survey year, sex, and school grade, with psychosomatic concerns
eTable 3. Associations of zBMI categories, survey year, sex, and school grade, with psychosomatic concerns, without imputation
eTable 4. Associations of zBMI categories, survey year, sex, and school grade, with psychosomatic concerns, cross validation
eFigure 1. Generalised additive models of psychosomatic concerns as a function of zBMI, by survey year by sex by grade
eFigure 2. Joint associations and their temporal trends of zBMI and sub-group variables with psychosomatic concerns
eFigure 3. Generalised additive models of psychosomatic concerns as a function of zBMI, by survey year, without imputation
eFigure 4. Generalised additive models of psychosomatic concerns as a function of zBMI, by survey year by sex, without imputation
eFigure 5. Generalised additive models of psychosomatic concerns as a function of zBMI, by survey year by grade, without imputation
eFigure 6. Generalised additive models of psychosomatic concerns as a function of zBMI, by survey year by sex by grade, without imputation
eFigure 7. Joint associations and their temporal trends of zBMI and sub-group variables with psychosomatic concerns, without imputation

This supplemental material has been provided by the authors to give readers additional information about their work.

eTable 1. Distribution of missing values by zBMI categories. Balance between cases with and without missing values was assessed by standardized mean difference (SMD). A absolute value of SMD > 0.1 indicates an imbalance between groups.

Variable	Thinness			Underweight			Normal weight			Overweight			Obese		
	No missing values	Had missing values	SMD	No missing values	Had missing values	SMD	No missing values	Had missing values	SMD	No missing values	Had missing values	SMD	No missing values	Had missing values	SMD
**Age (years)**	13.21 (1.56)	13.26 (1.59)	-0.034	13.56 (1.63)	13.53 (1.62)	0.021	13.77 (1.64)	13.41 (1.62)	**0.224**	13.52 (1.63)	13.00 (1.57)	**0.326**	13.34 (1.64)	12.88 (1.58)	**0.288**
**Sex (=female)**	17982 (53.3%)	11006 (52.3%)	0.020	59052 (59.2%)	26159 (57.6%)	0.034	231789 (53.3%)	102564 (51.3%)	0.040	41990 (41.2%)	21009 (40.3%)	0.019	10382 (33.7%)	5652 (33.8%)	-0.003
**School grade**															
Grade 5 (primary school)	13779 (40.9%)	8620 (41.0%)		32411 (32.5%)	15524 (34.2%)		120982 (27.8%)	74380 (37.2%)		34047 (33.4%)	25419 (48.7%)		12085 (39.2%)	9000 (53.8%)	
Grade 7 (middle school)	12515 (37.1%)	7523 (35.8%)	0.034	35619 (35.7%)	16363 (36.0%)	0.046	149417 (34.3%)	70373 (35.2%)	**0.245**	36221 (35.5%)	16605 (31.8%)	**0.344**	10189 (33.0%)	4641 (27.8%)	**0.310**
Grade 9 (high school)	7435 (22.0%)	4895 (23.3%)		31710 (31.8%)	13565 (29.8%)		164748 (37.9%)	55298 (27.6%)		31709 (31.1%)	10152 (19.5%)		8570 (27.8%)	3074 (18.4%)	
**Living with parents**															
Both	25559 (75.8%)	15477 (73.6%)		76486 (76.7%)	33705 (74.2%)		330626 (76.0%)	146012 (73.0%)		75742 (74.3%)	37237 (71.4%)		21953 (71.2%)	11634 (69.6%)	
One of parents	7063 (20.9%)	4465 (21.2%)	0.097	20586 (20.6%)	9508 (20.9%)	**0.119**	92389 (21.2%)	43373 (21.7%)	**0.132**	23020 (22.6%)	11753 (22.5%)	**0.142**	7723 (25.0%)	3927 (23.5%)	**0.140**
Neither	1107 (3.3%)	1096 (5.2%)		2668 (2.7%)	2239 (4.9%)		12132 (2.8%)	10666 (5.3%)		3215 (3.2%)	3186 (6.1%)		1168 (3.8%)	1154 (6.9%)	
**Sibling presence (= TRUE)**	29159 (86.5%)	18256 (86.8%)	-0.010	86314 (86.5%)	39395 (86.7%)	-0.004	372922 (85.7%)	171664 (85.8%)	-0.003	85606 (83.9%)	44262 (84.8%)	-0.024	25496 (82.7%)	14097 (84.3%)	-0.045
**Academic pressure**															
Not at all	7751 (23.0%)	4884 (23.2%)		20734 (20.8%)	9827 (21.6%)		86151 (19.8%)	43235 (21.6%)		20597 (20.2%)	11857 (22.7%)		6056 (19.6%)	3842 (23.0%)	
A little	14729 (43.7%)	8990 (42.7%)	0.053	45762 (45.9%)	19453 (42.8%)	0.087	195575 (44.9%)	85587 (42.8%)	0.079	44723 (43.9%)	22118 (42.4%)	0.077	13110 (42.5%)	7010 (41.9%)	0.091
Some	7498 (22.2%)	4483 (21.3%)		23171 (23.2%)	10494 (23.1%)		106211 (24.4%)	45953 (23.0%)		24716 (24.2%)	11611 (22.3%)		7525 (24.4%)	3649 (21.8%)	
A lot	3751 (11.1%)	2681 (12.7%)		10073 (10.1%)	5678 (12.5%)		47210 (10.8%)	25276 (12.6%)		11941 (11.7%)	6590 (12.6%)		4153 (13.5%)	2214 (13.2%)	
**Been bullied**															
Haven't	22663 (67.2%)	14296 (68.0%)		71533 (71.7%)	31866 (70.1%)		316622 (72.8%)	137925 (68.9%)		69449 (68.1%)	34139 (65.4%)		18985 (61.6%)	10447 (62.5%)	
Once or twice	6516 (19.3%)	4016 (19.1%)		18014 (18.1%)	8545 (18.8%)		75696 (17.4%)	37780 (18.9%)		19255 (18.9%)	10463 (20.1%)		6367 (20.6%)	3359 (20.1%)	
2-3 times per month	1768 (5.2%)	1023 (4.9%)	0.021	4087 (4.1%)	1987 (4.4%)	0.040	17424 (4.0%)	9498 (4.7%)	0.093	5206 (5.1%)	2930 (5.6%)	0.060	1969 (6.4%)	1057 (6.3%)	0.020
Once per week	1125 (3.3%)	682 (3.2%)		2664 (2.7%)	1244 (2.7%)		11164 (2.6%)	6071 (3.0%)		3391 (3.3%)	1843 (3.5%)		1334 (4.3%)	702 (4.2%)	
Several times per week	1657 (4.9%)	1021 (4.9%)		3442 (3.5%)	1810 (4.0%)		14241 (3.3%)	8777 (4.4%)		4676 (4.6%)	2801 (5.4%)		2189 (7.1%)	1150 (6.9%)	
**Family Affluence Scale**	5.40 (2.19)	5.40 (2.20)	-0.001	5.51 (2.11)	5.46 (2.17)	0.026	5.52 (2.06)	5.45 (2.13)	0.031	5.44 (2.01)	5.41 (2.10)	0.011	5.33 (1.99)	5.34 (2.12)	-0.004
**Screen time (hours per day)**	5.95 (3.80)	6.09 (3.92)	-0.037	5.82 (3.60)	6.07 (3.81)	-0.070	5.93 (3.58)	6.17 (3.82)	-0.065	6.28 (3.72)	6.29 (3.90)	-0.001	6.71 (3.95)	6.59 (4.09)	0.028
**Physical activity (days per week)**	4.12 (2.15)	4.04 (2.19)	0.035	4.07 (2.09)	3.99 (2.16)	0.034	4.10 (2.06)	4.03 (2.14)	0.034	3.95 (2.05)	3.98 (2.15)	-0.017	3.68 (2.09)	3.92 (2.20)	-0.101
**Psychosomatic concerns**	8.20 (6.69)	8.21 (6.79)	-0.002	7.94 (6.36)	8.12 (6.63)	-0.028	8.09 (6.41)	8.20 (6.70)	-0.016	8.29 (6.59)	8.12 (6.76)	0.026	8.92 (7.03)	8.33 (7.06)	0.083

© 2024 Chen S et al. *JAMA Psychiatry.*

eTable 2. **Associations of survey year, sex, and school grade, with psychosomatic concerns.** Data was presented as coefficient and its 95% confidence interval (CI), which were extracted from multi-level generalized additive model, with psychosomatic complaints as the outcome and interested variable as the exposure, as well as the random intercept and random slope for exposure at the level of classroom, school, and country.

**Association between survey year and psychosomatic concerns**						
	**Confounders**	**Survey year**				
		2002	2006	2010	2014	2018
Model 2	Univariable model	Ref	0.19(0.11, 0.26)	0.14(0.07, 0.22)	0.48(0.40, 0.56)	0.82(0.74, 0.89)
**Association between sex and psychosomatic concerns**						
	**Confounders**	**Sex**				
		Males	Females			
Model 3	Univariable model	Ref	2.27(2.25, 2.30)			
**Association between school grade and psychosomatic concerns**						
	**Confounders**	**School grade**				
		Primary school	Middle school	High school		
Model 4.1	Univariable model	Ref	1.17(1.14, 1.20)	2.18(2.15, 2.21)		
Model 4.2	Model 4.1 + sex	Ref	1.17(1.14, 1.20)	2.16(2.13, 2.19)		
Model 4.3	Model 4.2 + living with parents	Ref	1.15(1.12, 1.18)	2.12(2.09, 2.15)		
Model 4.4	model 4.3 + sibling presence	Ref	1.15(1.12, 1.18)	2.12(2.09, 2.16)		
Model 4.5	Model 4.4 + family affluence	Ref	1.15(1.12, 1.18)	2.12(2.09, 2.15)		

© 2024 Chen S et al. *JAMA Psychiatry.*

eTable 3. Associations of zBMI categories, survey year, sex, and school grade, with psychosomatic concerns, without imputation. Data was presented as coefficient and its 95% confidence interval (CI), which were extracted from multi-level generalized additive model, with psychosomatic concerns as the outcome and interested variable as the exposure, as well as the random intercept and random slope for exposure at the level of classroom, school, and country.

**Association between zBMI categories and psychosomatic concerns**						
	**Confounders**	**zBMI categories**				
		Thinness	Underweight	Normal weight	Overweight	Obese
Model 1.1	Univariable model	0.07(0.00, 0.14)	-0.14(-0.19, -0.10)	Ref	0.17(0.13, 0.21)	0.73(0.66, 0.81)
Model 1.2	Model 1.1 + survey year	0.07(0.00, 0.14)	-0.14(-0.19, -0.10)	Ref	0.15(0.11, 0.19)	0.70(0.62, 0.77)
Model 1.3	Model 1.2 + sex	0.08(0.01, 0.15)	-0.30(-0.34, -0.25)	Ref	0.46(0.42, 0.50)	1.20(1.13, 1.27)
Model 1.4	Model 1.3 + school grade	0.38(0.31, 0.45)	-0.18(-0.22, -0.14)	Ref	0.60(0.56, 0.65)	1.46(1.39, 1.53)
Model 1.5	Model 1.4 + living with parents	0.38(0.31, 0.45)	-0.17(-0.21, -0.13)	Ref	0.58(0.54, 0.62)	1.41(1.33, 1.48)
Model 1.6	model 1.5 + sibling presence	0.38(0.31, 0.45)	-0.17(-0.21, -0.13)	Ref	0.58(0.54, 0.62)	1.41(1.34, 1.48)
Model 1.7	Model 1.6 + family affluence	0.37(0.31, 0.44)	-0.17(-0.21, -0.13)	Ref	0.57(0.53, 0.61)	1.39(1.32, 1.46)
Model 1.8	Model 1.7 + screen time	0.36(0.29, 0.43)	-0.16(-0.20, -0.12)	Ref	0.52(0.47, 0.56)	1.25(1.18, 1.33)
Model 1.9	Model 1.8 + physical activity	0.35(0.28, 0.42)	-0.17(-0.21, -0.12)	Ref	0.47(0.43, 0.51)	1.15(1.08, 1.22)
Model 1.10	Model 1.9 + experience of been bullying	0.22(0.15, 0.29)	-0.17(-0.21, -0.13)	Ref	0.33(0.29, 0.37)	0.79(0.72, 0.86)
Model 1.11	model 1.10 + academic pressure	0.17(0.11, 0.23)	-0.17(-0.21, -0.13)	Ref	0.31(0.27, 0.35)	0.73(0.66, 0.80)
**Association between survey year and psychosomatic concerns**						
	**Confounders**	**Survey year**				
		2002	2006	2010	2014	2018
Model 2	Univariable model	Ref	0.21(0.13, 0.30)	0.14(0.06, 0.22)	0.53(0.44, 0.61)	0.92(0.84, 1.01)
**Association between sex and psychosomatic concerns**						
	**Confounders**	**Sex**				
		Males	Females			
Model 3	Univariable model	Ref	2.46(2.43, 2.49)			
**Association between school grade and psychosomatic concerns**						
	**Confounders**	**School grade**				
		Primary school	Middle school	High school		
Model 4.1	Univariable model	Ref	1.22(1.19, 1.26)	2.23(2.19, 2.27)		
Model 4.2	Model 4.1 + sex	Ref	1.21(1.17, 1.25)	2.19(2.16, 2.23)		
Model 4.3	Model 4.2 + living with parents	Ref	1.18(1.15, 1.22)	2.15(2.11, 2.19)		
Model 4.4	model 4.3 + sibling presence	Ref	1.18(1.15, 1.22)	2.15(2.11, 2.19)		
Model 4.5	Model 4.4 + family affluence	Ref	1.19(1.15, 1.22)	2.15(2.11, 2.19)		

eTable 4. Associations of zBMI categories, survey year, sex, and school grade, with psychosomatic concerns, cross validation. Data was presented as coefficient

© 2024 Chen S et al. *JAMA Psychiatry.*

and its 95% confidence interval (CI), which were extracted from multi-level generalized additive model, with psychosomatic concerns as the outcome and interested variable as the exposure, as well as the random intercept and random slope for exposure at the level of classroom, school, and country.

**Association between zBMI categories and psychosomatic concerns**						
	**Confounders**	**zBMI categories**				
		Thinness	Underweight	Normal weight	Overweight	Obese
Full dataset	Model 1.11 in Table 2	0.14(0.08, 0.19)	-0.18(-0.22, -0.15)	Ref	0.27(0.24, 0.30)	0.62(0.56, 0.67)
Sub dataset 1	Model 1.11 in Table 2	0.13(0.02, 0.25)	-0.17(-0.24, -0.09)	Ref	0.31(0.24, 0.39)	0.59(0.47, 0.71)
Sub dataset 2	Model 1.11 in Table 2	0.16(0.04, 0.27)	-0.18(-0.25, -0.10)	Ref	0.32(0.25, 0.40)	0.57(0.45, 0.69)
Sub dataset 3	Model 1.11 in Table 2	0.16(0.05, 0.28)	-0.23(-0.30, -0.15)	Ref	0.25(0.18, 0.32)	0.68(0.56, 0.81)
Sub dataset 4	Model 1.11 in Table 2	0.20(0.09, 0.32)	-0.19(-0.27, -0.12)	Ref	0.19(0.11, 0.26)	0.68(0.56, 0.81)
Sub dataset 5	Model 1.11 in Table 2	0.13(0.02, 0.25)	-0.16(-0.23, -0.08)	Ref	0.29(0.22, 0.36)	0.58(0.46, 0.71)

**Association between survey year and psychosomatic concerns**						
		**Survey year**				
	**Confounders**	2002	2006	2010	2014	2018
Full dataset	Model 2 in Table 2	Ref	0.19(0.11, 0.26)	0.14(0.07, 0.22)	0.48(0.40, 0.56)	0.82(0.74, 0.89)
Sub dataset 1	Model 2 in Table 2	Ref	0.16(0.06, 0.26)	0.13(0.03, 0.23)	0.46(0.35, 0.56)	0.83(0.73, 0.93)
Sub dataset 2	Model 2 in Table 2	Ref	0.13(0.03, 0.23)	0.15(0.04, 0.25)	0.48(0.38, 0.58)	0.80(0.70, 0.90)
Sub dataset 3	Model 2 in Table 2	Ref	0.17(0.07, 0.28)	0.11(0.01, 0.23)	0.50(0.40, 0.61)	0.83(0.73, 0.93)
Sub dataset 4	Model 2 in Table 2	Ref	0.25(0.15, 0.35)	0.16(0.06, 0.27)	0.48(0.38, 0.59)	0.81(0.70, 0.91)
Sub dataset 5	Model 2 in Table 2	Ref	0.16(0.06, 0.26)	0.10(0.00, 0.20)	0.40(0.30, 0.50)	0.80(0.70, 0.90)
**Association between sex and psychosomatic concerns**						
		**Sex**				
	**Confounders**	Males	Females			
Full dataset	Model 3 in Table 2	Ref	2.27(2.25, 2.30)			
Sub dataset 1	Model 3 in Table 2	Ref	2.33(2.28, 2.39)			
Sub dataset 2	Model 3 in Table 2	Ref	2.26(2.20, 2.31)			
Sub dataset 3	Model 3 in Table 2	Ref	2.25(2.20, 2.31)			
Sub dataset 4	Model 3 in Table 2	Ref	2.25(2.20, 2.30)			
Sub dataset 5	Model 3 in Table 2	Ref	2.27(2.21, 2.32)			
**Association between school grade and psychosomatic concerns**						
		**School grade**				
		Primary school	Middle school	High school		
Full dataset	Model 4.5 in Table 2	Ref	1.15(1.12, 1.18)	2.12(2.09, 2.15)		
Sub dataset 1	Model 4.5 in Table 2	Ref	1.13(1.06, 1.19)	2.09(2.02, 2.16)		

© 2024 Chen S et al. *JAMA Psychiatry.*

Sub dataset 2	Model 4.5 in Table 2	Ref	1.11(1.05, 1.18)	2.08(2.01, 2.14)		
Sub dataset 3	Model 4.5 in Table 2	Ref	1.15(1.09, 1.22)	2.12(2.05, 2.19)		
Sub dataset 4	Model 4.5 in Table 2	Ref	1.18(1.11, 1.24)	2.16(2.09, 2.23)		
Sub dataset 5	Model 4.5 in Table 2	Ref	1.17(1.11, 1.24)	2.18(2.11, 2.25)		

© 2024 Chen S et al. *JAMA Psychiatry.*

eTable 5. Joint associations and their temporal trends of zBMI and sub-group variables with psychosomatic concerns, cross validation.

**Panel A, associations between zBMI (ref = normal weight) and psychosomatic concerns, which extracted from model 1.11 in Table 2;**						
	Full dataset	Sub dataset 1	Sub dataset 2	Sub dataset 3	Sub dataset 4	Sub dataset 5
zBMI (= Thinness)	0.14(0.08, 0.19)	0.13(0.02, 0.25)	0.16(0.04, 0.27)	0.16(0.05, 0.28)	0.20(0.09, 0.32)	0.13(0.02, 0.25)
zBMI (= Underweight)	-0.18(-0.22, -0.15)	-0.17(-0.24, -0.09)	-0.18(-0.25, -0.10)	-0.23(-0.30, -0.15)	-0.19(-0.27, -0.12)	-0.16(-0.23, -0.08)
zBMI (= Normal weight)	Ref	Ref	Ref	Ref	Ref	Ref
zBMI (= Overweight)	0.27(0.24, 0.30)	0.31(0.24, 0.39)	0.32(0.25, 0.40)	0.25(0.18, 0.32)	0.19(0.11, 0.26)	0.29(0.22, 0.36)
zBMI (= Obese)	0.62(0.56, 0.67)	0.59(0.47, 0.71)	0.57(0.45, 0.69)	0.68(0.56, 0.81)	0.68(0.56, 0.81)	0.58(0.46, 0.71)
**Panel B, the temporal trend on the association between zBMI and psychosomatic concerns, which extracted from model 1.11 in Table 2 + zBMI categories (ref = normal weight) x survey year (ref = 2002);**						
zBMI (= Normal weight) x survey year (= 2002)	Ref	Ref	Ref	Ref	Ref	Ref
zBMI (= Thinness) x survey year (= 2006)	0.05(-0.13, 0.22)	-0.03(-0.42, 0.35)	-0.16(-0.54, 0.22)	0.10(-0.29, 0.48)	0.30(-0.08, 0.68)	0.02(-0.36, 0.41)
zBMI (= Underweight) x survey year (= 2006)	-0.07(-0.18, 0.04)	-0.14(-0.38, 0.11)	-0.10(-0.35, 0.14)	-0.12(-0.36, 0.13)	0.02(-0.23, 0.27)	0.05(-0.20, 0.30)
zBMI (= Overweight) x survey year (= 2006)	-0.09(-0.20, 0.03)	-0.24(-0.50, 0.01)	-0.24(-0.49, 0.02)	-0.07(-0.32, 0.19)	0.03(-0.22, 0.29)	0.00(-0.25, 0.26)
zBMI (= Obese) x survey year (= 2006)	0.01(-0.19, 0.21)	0.08(-0.37, 0.53)	0.14(-0.31, 0.58)	-0.16(-0.60, 0.28)	-0.14(-0.59, 0.31)	-0.10(-0.54, 0.34)
zBMI (= Thinness) x survey year (= 2010)	0.15(-0.02, 0.32)	-0.01(-0.39, 0.38)	-0.12(-0.51, 0.26)	0.25(-0.14, 0.63)	0.15(-0.23, 0.54)	0.51(0.13, 0.89)
zBMI (= Underweight) x survey year (= 2010)	-0.02(-0.13, 0.09)	-0.23(-0.48, 0.02)	0.16(-0.08, 0.41)	-0.06(-0.30, 0.19)	0.02(-0.23, 0.27)	0.10(-0.15, 0.35)
zBMI (= Overweight) x survey year (= 2010)	-0.07(-0.18, 0.04)	-0.25(-0.50, 0.00)	-0.12(-0.37, 0.13)	-0.13(-0.38, 0.12)	0.08(-0.17, 0.33)	-0.08(-0.33, 0.17)
zBMI (= Obese) x survey year (= 2010)	0.09(-0.11, 0.28)	-0.00(-0.44, 0.43)	0.22(-0.21, 0.65)	0.20(-0.22, 0.63)	-0.13(-0.56, 0.30)	-0.12(-0.55, 0.30)
zBMI (= Thinness) x survey year (= 2014)	0.06(-0.11, 0.23)	0.04(-0.34, 0.41)	-0.19(-0.56, 0.18)	-0.02(-0.39, 0.36)	0.35(-0.03, 0.73)	0.09(-0.29, 0.47)
zBMI (= Underweight) x survey year (= 2014)	0.05(-0.06, 0.16)	-0.08(-0.32, 0.17)	0.01(-0.24, 0.25)	0.05(-0.20, 0.30)	0.23(-0.02, 0.47)	0.10(-0.15, 0.34)
zBMI (= Overweight) x survey year (= 2014)	-0.15(-0.26, -0.04)	-0.34(-0.59, -0.09)	-0.25(-0.50, 0.00)	-0.26(-0.51, -0.01)	0.00(-0.25, 0.25)	-0.21(-0.46, 0.04)
zBMI (= Obese) x survey year (= 2014)	-0.01(-0.20, 0.18)	-0.32(-0.76, 0.12)	0.24(-0.19, 0.67)	-0.23(-0.66, 0.19)	0.08(-0.36, 0.51)	-0.04(-0.47, 0.39)
zBMI (= Thinness) x survey year (= 2018)	0.25(0.09, 0.41)	0.39(0.03, 0.75)	0.22(-0.13, 0.58)	0.36(0.00, 0.73)	0.54(0.17, 0.90)	0.16(-0.20, 0.52)
zBMI (= Underweight) x survey year (= 2018)	-0.00(-0.11, 0.10)	-0.16(-0.40, 0.09)	0.03(-0.21, 0.27)	0.02(-0.22, 0.26)	0.07(-0.17, 0.31)	0.05(-0.19, 0.30)
zBMI (= Overweight) x survey year (= 2018)	-0.07(-0.18, 0.04)	-0.23(-0.47, 0.01)	-0.19(-0.43, 0.06)	-0.11(-0.36, 0.14)	0.19(-0.05, 0.43)	-0.07(-0.31, 0.17)
zBMI (= Obese) x survey year (= 2018)	0.03(-0.16, 0.21)	-0.15(-0.57, 0.28)	-0.01(-0.43, 0.40)	0.02(-0.40, 0.43)	0.03(-0.39, 0.45)	0.04(-0.37, 0.46)
**Panel C, the effect modification of sex on the association between zBMI and psychosomatic concerns, which extracted from model 1.11 in Table 2 + zBMI categories (ref = normal weight) x sex (ref = males);**						
zBMI (= Normal weight) x sex (=males)	Ref	Ref	Ref	Ref	Ref	Ref
zBMI (= Thinness) x sex (= females)	-0.47(-0.58, -0.37)	-0.24(-0.47, -0.01)	-0.42(-0.65, -0.19)	-0.61(-0.84, -0.39)	-0.63(-0.86, -0.40)	-0.46(-0.69, -0.23)
zBMI (= Underweight) x sex (= females)	-0.33(-0.40, -0.27)	-0.30(-0.45, -0.14)	-0.30(-0.45, -0.14)	-0.36(-0.51, -0.21)	-0.37(-0.52, -0.22)	-0.35(-0.50, -0.20)
zBMI (= Overweight) x sex (= females)	0.08(0.01, 0.14)	0.23(0.08, 0.38)	0.16(0.01, 0.31)	-0.09(-0.23, 0.06)	0.07(-0.08, 0.21)	0.06(-0.08, 0.21)
zBMI (= Obese) x sex (= females)	-0.14(-0.25, -0.02)	-0.27(-0.53, -0.01)	-0.07(-0.33, 0.19)	-0.34(-0.60, -0.09)	0.10(-0.16, 0.36)	-0.12(-0.38, 0.13)
**Panel D, the effect modification of school grade on the association between zBMI and psychosomatic concerns, which extracted from model 1.11 in Table 2 + zBMI categories (ref = normal weight) x school grade(ref = primary school);**						
zBMI (= Normal weight) x school grade(=primary school)	Ref	Ref	Ref	Ref	Ref	Ref
zBMI (= Thinness) x school grade(=Grade 7)	-0.32(-0.44, -0.20)	-0.45(-0.71, -0.19)	-0.30(-0.57, -0.04)	-0.33(-0.59, -0.07)	-0.08(-0.35, 0.18)	-0.43(-0.69, -0.16)
zBMI (= Underweight) x school grade(=Grade 7)	-0.12(-0.20, -0.04)	-0.25(-0.43, -0.07)	-0.19(-0.37, -0.01)	0.02(-0.16, 0.20)	-0.11(-0.29, 0.07)	-0.10(-0.29, 0.08)
zBMI (= Overweight) x school grade(=Grade 7)	-0.03(-0.11, 0.05)	-0.01(-0.18, 0.16)	0.08(-0.10, 0.25)	-0.02(-0.19, 0.15)	-0.09(-0.26, 0.09)	-0.11(-0.28, 0.06)
zBMI (= Obese) x school grade(=Grade 7)	-0.18(-0.31, -0.05)	-0.31(-0.60, -0.02)	-0.10(-0.38, 0.19)	-0.13(-0.42, 0.16)	-0.05(-0.34, 0.24)	-0.32(-0.61, -0.03)
zBMI (= Thinness) x school grade(=Grade 9)	-0.26(-0.39, -0.12)	-0.49(-0.79, -0.19)	-0.21(-0.51, 0.08)	-0.19(-0.49, 0.11)	0.12(-0.18, 0.42)	-0.53(-0.82, -0.23)
zBMI (= Underweight) x school grade(=Grade 9)	0.12(0.03, 0.20)	0.22(0.03, 0.40)	-0.02(-0.21, 0.17)	0.18(0.00, 0.37)	0.34(0.15, 0.52)	-0.08(-0.27, 0.10)
zBMI (= Overweight) x school grade(=Grade 9)	-0.12(-0.20, -0.04)	-0.19(-0.38, -0.01)	-0.05(-0.23, 0.13)	-0.06(-0.24, 0.12)	-0.21(-0.39, -0.03)	-0.21(-0.39, -0.02)
zBMI (= Obese) x school grade(=Grade 9)	-0.29(-0.43, -0.16)	-0.31(-0.61, -0.00)	-0.39(-0.70, -0.08)	-0.25(-0.56, 0.06)	-0.12(-0.42, 0.19)	-0.39(-0.70, -0.08)

© 2024 Chen S et al. *JAMA Psychiatry.*

**Panel E, the effect modification of sex on the temporal trend of association between zBMI and psychosomatic concerns, which extracted from model 1.11 in Table 2 + zBMI categories (ref = normal weight) x survey year (ref = 2002) x sex(ref = males);**						
zBMI (= normal weight) x survey year (= 2002) x sex(= males)	Ref	Ref	Ref	Ref	Ref	Ref
zBMI (= Thinness) x survey year (= 2006) x sex (= females)	-0.05(-0.39, 0.30)	0.60(-0.18, 1.37)	0.20(-0.56, 0.96)	0.61(-0.16, 1.38)	-0.76(-1.52, 0.01)	-0.58(-1.36, 0.19)
zBMI (= Underweight) x survey year (= 2006) x sex (= females)	0.00(-0.22, 0.23)	0.04(-0.46, 0.54)	0.33(-0.17, 0.83)	-0.24(-0.75, 0.26)	0.12(-0.38, 0.62)	-0.18(-0.68, 0.32)
zBMI (= Overweight) x survey year (= 2006) x sex (= females)	-0.03(-0.26, 0.21)	-0.05(-0.57, 0.46)	0.40(-0.11, 0.91)	0.01(-0.51, 0.53)	-0.28(-0.80, 0.24)	-0.18(-0.70, 0.34)
zBMI (= Obese) x survey year (= 2006) x sex (= females)	-0.40(-0.82, 0.01)	-0.38(-1.32, 0.57)	0.10(-0.84, 1.03)	-0.90(-1.83, 0.03)	-0.34(-1.30, 0.61)	-0.24(-1.18, 0.69)
zBMI (= Thinness) x survey year (= 2010) x sex (= females)	0.04(-0.30, 0.38)	0.65(-0.12, 1.42)	0.19(-0.58, 0.95)	0.69(-0.08, 1.46)	-0.73(-1.51, 0.04)	-0.48(-1.25, 0.29)
zBMI (= Underweight) x survey year (= 2010) x sex (= females)	-0.11(-0.34, 0.11)	0.11(-0.39, 0.61)	0.03(-0.47, 0.53)	-0.39(-0.89, 0.11)	-0.10(-0.60, 0.40)	-0.11(-0.61, 0.39)
zBMI (= Overweight) x survey year (= 2010) x sex (= females)	0.12(-0.11, 0.35)	0.22(-0.29, 0.73)	0.44(-0.07, 0.95)	-0.03(-0.54, 0.48)	-0.24(-0.75, 0.27)	0.21(-0.30, 0.72)
zBMI (= Obese) x survey year (= 2010) x sex (= females)	-0.09(-0.49, 0.32)	-0.12(-1.04, 0.80)	0.50(-0.40, 1.41)	-0.63(-1.53, 0.26)	-0.42(-1.33, 0.48)	0.37(-0.52, 1.27)
zBMI (= Thinness) x survey year (= 2014) x sex (= females)	0.01(-0.33, 0.35)	0.57(-0.18, 1.32)	0.13(-0.62, 0.87)	0.56(-0.20, 1.31)	-0.67(-1.43, 0.09)	-0.41(-1.17, 0.35)
zBMI (= Underweight) x survey year (= 2014) x sex (= females)	-0.27(-0.50, -0.05)	-0.34(-0.84, 0.16)	-0.05(-0.54, 0.45)	-0.28(-0.78, 0.22)	-0.34(-0.84, 0.16)	-0.33(-0.83, 0.17)
zBMI (= Overweight) x survey year (= 2014) x sex (= females)	-0.02(-0.25, 0.21)	-0.07(-0.58, 0.43)	0.33(-0.18, 0.83)	-0.13(-0.64, 0.38)	-0.04(-0.54, 0.47)	-0.18(-0.69, 0.33)
zBMI (= Obese) x survey year (= 2014) x sex (= females)	0.11(-0.30, 0.51)	-0.21(-1.13, 0.71)	0.61(-0.29, 1.51)	0.03(-0.86, 0.93)	-0.28(-1.20, 0.64)	0.53(-0.37, 1.42)
zBMI (= Thinness) x survey year (= 2018) x sex (= females)	-0.03(-0.35, 0.30)	0.34(-0.38, 1.07)	0.34(-0.38, 1.06)	0.36(-0.37, 1.09)	-0.65(-1.38, 0.08)	-0.46(-1.18, 0.27)
zBMI (= Underweight) x survey year (= 2018) x sex (= females)	-0.14(-0.36, 0.07)	-0.01(-0.50, 0.48)	0.03(-0.46, 0.51)	-0.34(-0.83, 0.15)	-0.37(-0.85, 0.12)	-0.03(-0.52, 0.45)
zBMI (= Overweight) x survey year (= 2018) x sex (= females)	0.09(-0.13, 0.31)	-0.24(-0.73, 0.25)	0.48(-0.01, 0.98)	-0.15(-0.64, 0.35)	0.20(-0.29, 0.69)	0.07(-0.42, 0.57)
zBMI (= Obese) x survey year (= 2018) x sex (= females)	0.10(-0.29, 0.49)	-0.00(-0.89, 0.89)	0.68(-0.19, 1.56)	-0.18(-1.04, 0.69)	-0.22(-1.10, 0.66)	0.38(-0.49, 1.25)
**Panel F, the effect modification of school grade on the temporal trend of association between zBMI and psychosomatic concerns, which extracted from model 1.11 in Table 2 + zBMI categories (ref = normal weight) x survey year (ref = 2002) x school grade(ref = primary school);**						
zBMI (= normal weight) x survey year (= 2002) x school grade(= primary school)	Ref	Ref	Ref	Ref	Ref	Ref
zBMI (= Thinness) x survey year (= 2006) x school grade(=Grade 7)	-0.12(-0.51, 0.28)	0.81(-0.07, 1.69)	-0.34(-1.21, 0.53)	0.09(-0.78, 0.96)	-0.62(-1.49, 0.25)	-0.55(-1.43, 0.34)
zBMI (= Underweight) x survey year (= 2006) x school grade(=Grade 7)	0.13(-0.14, 0.40)	0.19(-0.41, 0.79)	-0.13(-0.73, 0.47)	0.21(-0.40, 0.81)	0.07(-0.54, 0.67)	0.34(-0.26, 0.93)
zBMI (= Overweight) x survey year (= 2006) x school grade(=Grade 7)	0.00(-0.27, 0.27)	-0.36(-0.96, 0.25)	-0.01(-0.60, 0.59)	0.37(-0.23, 0.97)	-0.10(-0.70, 0.51)	0.11(-0.50, 0.71)
zBMI (= Obese) x survey year (= 2006) x school grade(=Grade 7)	0.36(-0.11, 0.82)	-0.27(-1.32, 0.78)	0.74(-0.30, 1.79)	-0.63(-1.66, 0.40)	0.30(-0.75, 1.36)	1.01(-0.02, 2.03)
zBMI (= Thinness) x survey year (= 2010) x school grade(=Grade 7)	0.05(-0.34, 0.44)	0.81(-0.07, 1.69)	0.27(-0.60, 1.14)	-0.05(-0.93, 0.82)	-0.57(-1.44, 0.31)	-0.36(-1.23, 0.52)
zBMI (= Underweight) x survey year (= 2010) x school grade(=Grade 7)	-0.08(-0.34, 0.19)	0.51(-0.08, 1.11)	-0.05(-0.65, 0.55)	-0.21(-0.81, 0.39)	-0.55(-1.15, 0.06)	-0.06(-0.66, 0.54)
zBMI (= Overweight) x survey year (= 2010) x school grade(=Grade 7)	-0.07(-0.33, 0.20)	-0.58(-1.17, 0.01)	0.30(-0.29, 0.90)	-0.00(-0.59, 0.59)	-0.13(-0.73, 0.46)	0.13(-0.46, 0.72)
zBMI (= Obese) x survey year (= 2010) x school grade(=Grade 7)	0.02(-0.43, 0.47)	0.30(-0.72, 1.32)	0.12(-0.89, 1.12)	-0.98(-1.98, 0.01)	0.37(-0.64, 1.38)	1.08(0.09, 2.07)
zBMI (= Thinness) x survey year (= 2014) x school grade(=Grade 7)	0.05(-0.34, 0.43)	0.65(-0.21, 1.51)	0.54(-0.31, 1.39)	0.03(-0.83, 0.89)	-0.66(-1.53, 0.20)	-0.38(-1.25, 0.48)
zBMI (= Underweight) x survey year (= 2014) x school grade(=Grade 7)	-0.19(-0.45, 0.08)	0.05(-0.54, 0.65)	-0.02(-0.62, 0.57)	-0.82(-1.42, -0.22)	-0.18(-0.78, 0.42)	0.05(-0.54, 0.65)
zBMI (= Overweight) x survey year (= 2014) x school grade(=Grade 7)	-0.16(-0.42, 0.11)	-0.55(-1.14, 0.04)	0.11(-0.48, 0.70)	-0.01(-0.61, 0.58)	-0.27(-0.86, 0.32)	-0.05(-0.64, 0.55)
zBMI (= Obese) x survey year (= 2014) x school grade(=Grade 7)	-0.23(-0.68, 0.22)	-0.70(-1.72, 0.33)	0.19(-0.81, 1.19)	-1.18(-2.18, -0.19)	0.28(-0.74, 1.29)	0.27(-0.72, 1.27)
zBMI (= Thinness) x survey year (= 2018) x school grade(=Grade 7)	0.20(-0.17, 0.57)	0.82(-0.01, 1.65)	0.58(-0.24, 1.41)	0.43(-0.40, 1.26)	-0.47(-1.30, 0.36)	-0.58(-1.41, 0.24)
zBMI (= Underweight) x survey year (= 2018) x school grade(=Grade 7)	0.00(-0.26, 0.26)	0.15(-0.43, 0.72)	0.09(-0.49, 0.67)	-0.50(-1.08, 0.08)	0.37(-0.21, 0.96)	-0.11(-0.69, 0.47)
zBMI (= Overweight) x survey year (= 2018) x school grade(=Grade 7)	0.07(-0.19, 0.33)	-0.32(-0.89, 0.26)	0.08(-0.49, 0.66)	0.06(-0.52, 0.64)	0.42(-0.15, 0.99)	0.15(-0.42, 0.72)
zBMI (= Obese) x survey year (= 2018) x school grade(=Grade 7)	0.05(-0.39, 0.48)	-0.21(-1.20, 0.78)	-0.05(-1.02, 0.92)	-0.20(-1.16, 0.75)	0.93(-0.04, 1.91)	0.78(-0.19, 1.74)
zBMI (= Thinness) x survey year (= 2006) x school grade(=Grade 9)	-0.11(-0.56, 0.35)	0.03(-0.99, 1.04)	0.19(-0.80, 1.19)	-0.05(-1.07, 0.97)	0.08(-0.93, 1.09)	-0.85(-1.87, 0.18)
zBMI (= Underweight) x survey year (= 2006) x school grade(=Grade 9)	-0.03(-0.30, 0.25)	-0.02(-0.64, 0.60)	-0.08(-0.69, 0.54)	0.06(-0.56, 0.67)	0.10(-0.51, 0.71)	-0.18(-0.80, 0.44)
zBMI (= Overweight) x survey year (= 2006) x school grade(=Grade 9)	-0.10(-0.38, 0.19)	-0.46(-1.10, 0.18)	0.10(-0.54, 0.73)	0.37(-0.28, 1.01)	-0.52(-1.16, 0.12)	0.10(-0.54, 0.74)
zBMI (= Obese) x survey year (= 2006) x school grade(=Grade 9)	-0.02(-0.53, 0.49)	0.42(-0.72, 1.55)	0.02(-1.13, 1.16)	-1.35(-2.48, -0.22)	0.00(-1.13, 1.14)	0.89(-0.25, 2.02)
zBMI (= Thinness) x survey year (= 2010) x school grade(=Grade 9)	0.02(-0.44, 0.47)	-0.05(-1.06, 0.96)	0.23(-0.78, 1.24)	-0.05(-1.07, 0.96)	0.32(-0.72, 1.36)	-0.45(-1.47, 0.56)
zBMI (= Underweight) x survey year (= 2010) x school grade(=Grade 9)	0.18(-0.09, 0.46)	0.61(-0.01, 1.23)	0.38(-0.24, 1.00)	0.60(-0.01, 1.22)	-0.54(-1.15, 0.08)	-0.21(-0.82, 0.40)

© 2024 Chen S et al. *JAMA Psychiatry.*

zBMI (= Overweight) x survey year (= 2010) x school grade(=Grade 9)	0.17(-0.11, 0.45)	-0.12(-0.74, 0.51)	0.26(-0.37, 0.89)	0.56(-0.08, 1.19)	-0.28(-0.90, 0.34)	0.51(-0.12, 1.13)
zBMI (= Obese) x survey year (= 2010) x school grade(=Grade 9)	0.01(-0.48, 0.50)	0.79(-0.30, 1.88)	-0.75(-1.85, 0.36)	-1.03(-2.13, 0.06)	0.93(-0.16, 2.02)	0.13(-0.97, 1.23)
zBMI (= Thinness) x survey year (= 2014) x school grade(=Grade 9)	0.38(-0.06, 0.83)	0.02(-0.96, 1.01)	1.35(0.37, 2.34)	0.09(-0.91, 1.08)	0.83(-0.17, 1.84)	-0.44(-1.44, 0.57)
zBMI (= Underweight) x survey year (= 2014) x school grade(=Grade 9)	-0.08(-0.35, 0.19)	0.22(-0.39, 0.83)	0.20(-0.41, 0.81)	-0.22(-0.84, 0.39)	-0.26(-0.87, 0.35)	-0.35(-0.96, 0.26)
zBMI (= Overweight) x survey year (= 2014) x school grade(=Grade 9)	-0.18(-0.46, 0.10)	-0.20(-0.83, 0.43)	0.27(-0.36, 0.89)	-0.38(-1.01, 0.25)	-0.59(-1.21, 0.03)	0.31(-0.31, 0.94)
zBMI (= Obese) x survey year (= 2014) x school grade(=Grade 9)	-0.20(-0.69, 0.29)	-0.22(-1.32, 0.87)	-0.32(-1.42, 0.78)	-1.04(-2.12, 0.04)	0.70(-0.41, 1.81)	0.05(-1.03, 1.14)
zBMI (= Thinness) x survey year (= 2018) x school grade(=Grade 9)	0.26(-0.16, 0.69)	0.18(-0.78, 1.13)	0.69(-0.25, 1.62)	0.47(-0.49, 1.44)	0.85(-0.11, 1.81)	-0.95(-1.90, 0.01)
zBMI (= Underweight) x survey year (= 2018) x school grade(=Grade 9)	0.13(-0.14, 0.39)	0.59(-0.01, 1.20)	0.10(-0.50, 0.70)	0.16(-0.44, 0.76)	-0.11(-0.71, 0.48)	-0.14(-0.73, 0.46)
zBMI (= Overweight) x survey year (= 2018) x school grade(=Grade 9)	0.17(-0.10, 0.45)	-0.03(-0.64, 0.58)	0.16(-0.46, 0.77)	0.39(-0.23, 1.01)	0.07(-0.54, 0.67)	0.36(-0.26, 0.97)
zBMI (= Obese) x survey year (= 2018) x school grade(=Grade 9)	0.03(-0.45, 0.50)	0.19(-0.87, 1.24)	-0.63(-1.69, 0.42)	-1.00(-2.06, 0.06)	0.89(-0.18, 1.96)	1.05(-0.01, 2.11)
**Panel G, the joint effect modification of sex and school grade on the associations between zBMI and psychosomatic concerns, which extracted from model 1.11 in Table 2 + zBMI categories (ref = normal weight) x sex (ref = males) x school grade(ref = primary school);**						
zBMI (= normal weight) x sex (= males) x school grade(= primary school)	Ref	Ref	Ref	Ref	Ref	Ref
zBMI (= Thinness) x sex (= females)) x school grade(=Grade 7)	-0.28(-0.52, -0.05)	-0.73(-1.26, -0.21)	0.14(-0.39, 0.66)	0.16(-0.36, 0.68)	-0.62(-1.14, -0.09)	-0.55(-1.08, -0.02)
zBMI (= Underweight) x sex (= females)) x school grade(=Grade 7)	-0.30(-0.46, -0.13)	-0.50(-0.87, -0.14)	-0.38(-0.74, -0.01)	-0.15(-0.52, 0.21)	-0.18(-0.55, 0.18)	-0.48(-0.84, -0.11)
zBMI (= Overweight) x sex (= females)) x school grade(=Grade 7)	0.04(-0.11, 0.20)	-0.03(-0.38, 0.32)	0.13(-0.22, 0.48)	0.34(-0.01, 0.69)	0.04(-0.32, 0.39)	-0.25(-0.60, 0.10)
zBMI (= Obese) x sex (= females)) x school grade(=Grade 7)	0.12(-0.15, 0.40)	-0.16(-0.76, 0.45)	0.52(-0.09, 1.12)	-0.25(-0.85, 0.35)	-0.00(-0.61, 0.60)	0.49(-0.11, 1.10)
zBMI (= Thinness) x sex (= females)) x school grade(=Grade 9)	-0.14(-0.41, 0.13)	-0.41(-1.00, 0.19)	-0.03(-0.62, 0.56)	0.33(-0.27, 0.93)	-0.07(-0.67, 0.53)	-0.54(-1.13, 0.06)
zBMI (= Underweight) x sex (= females)) x school grade(=Grade 9)	-0.11(-0.28, 0.05)	-0.12(-0.50, 0.26)	0.14(-0.23, 0.52)	-0.11(-0.49, 0.27)	-0.04(-0.41, 0.33)	-0.34(-0.72, 0.03)
zBMI (= Overweight) x sex (= females)) x school grade(=Grade 9)	0.13(-0.03, 0.30)	0.00(-0.37, 0.37)	0.26(-0.11, 0.63)	0.36(-0.01, 0.73)	0.24(-0.13, 0.61)	-0.24(-0.61, 0.13)
zBMI (= Obese) x sex (= females)) x school grade(=Grade 9)	-0.27(-0.56, 0.02)	-0.09(-0.73, 0.55)	-0.15(-0.79, 0.50)	-0.41(-1.05, 0.23)	-0.38(-1.03, 0.27)	-0.34(-0.99, 0.32)
**Panel H, the joint effect modification of sex and school grade on the temporal trend of associations between zBMI and psychosomatic concerns, which extracted from model 1.11 in Table 2 + zBMI categories (ref = normal weight) x survey year (ref = 2002) x sex (ref = males) x school grade(ref = primary school)**						
zBMI (= normal weight) x survey year (= 2002) x sex (= males) x school grade(= primary school)	Ref	Ref	Ref	Ref	Ref	Ref
zBMI (= Thinness) x survey year (= 2006) x sex (= females)) x school grade(=Grade 7)	-0.46(-1.24, 0.32)	-0.46(-2.22, 1.31)	-0.53(-2.27, 1.21)	-1.01(-2.74, 0.73)	0.20(-1.54, 1.94)	-0.43(-2.20, 1.34)
zBMI (= Underweight) x survey year (= 2006) x sex (= females)) x school grade(=Grade 7)	0.02(-0.52, 0.56)	-0.02(-1.23, 1.19)	0.10(-1.12, 1.31)	0.72(-0.49, 1.94)	-0.27(-1.49, 0.94)	-0.59(-1.79, 0.61)
zBMI (= Overweight) x survey year (= 2006) x sex (= females)) x school grade(=Grade 7)	0.00(-0.54, 0.55)	-0.44(-1.66, 0.78)	0.85(-0.36, 2.06)	-0.15(-1.37, 1.07)	-0.29(-1.51, 0.93)	0.03(-1.19, 1.25)
zBMI (= Obese) x survey year (= 2006) x sex (= females)) x school grade(=Grade 7)	-0.31(-1.29, 0.67)	-0.11(-2.32, 2.09)	1.24(-0.95, 3.42)	-1.04(-3.21, 1.13)	-0.14(-2.38, 2.10)	-1.61(-3.78, 0.55)
zBMI (= Thinness) x survey year (= 2010) x sex (= females)) x school grade(=Grade 7)	-0.70(-1.48, 0.09)	-0.77(-2.52, 0.99)	-0.82(-2.56, 0.93)	1.02(-0.73, 2.77)	-1.61(-3.37, 0.14)	-1.20(-2.95, 0.56)
zBMI (= Underweight) x survey year (= 2010) x sex (= females)) x school grade(=Grade 7)	-0.19(-0.73, 0.35)	-0.38(-1.59, 0.83)	-0.03(-1.24, 1.17)	0.47(-0.74, 1.68)	-1.13(-2.34, 0.08)	-0.07(-1.28, 1.14)
zBMI (= Overweight) x survey year (= 2010) x sex (= females)) x school grade(=Grade 7)	0.24(-0.30, 0.78)	0.49(-0.70, 1.69)	1.11(-0.09, 2.30)	0.54(-0.66, 1.74)	-1.10(-2.31, 0.10)	0.12(-1.08, 1.31)
zBMI (= Obese) x survey year (= 2010) x sex (= females)) x school grade(=Grade 7)	-0.40(-1.34, 0.55)	0.33(-1.83, 2.49)	0.02(-2.09, 2.13)	-0.98(-3.08, 1.12)	-0.34(-2.49, 1.81)	-1.10(-3.18, 0.98)
zBMI (= Thinness) x survey year (= 2014) x sex (= females)) x school grade(=Grade 7)	-0.63(-1.40, 0.14)	-0.86(-2.57, 0.86)	-1.24(-2.94, 0.46)	1.41(-0.31, 3.13)	-1.16(-2.89, 0.57)	-1.17(-2.90, 0.56)
zBMI (= Underweight) x survey year (= 2014) x sex (= females)) x school grade(=Grade 7)	-0.31(-0.85, 0.22)	-0.18(-1.38, 1.02)	0.11(-1.09, 1.31)	0.61(-0.60, 1.81)	-1.59(-2.80, -0.38)	-0.65(-1.85, 0.55)
zBMI (= Overweight) x survey year (= 2014) x sex (= females)) x school grade(=Grade 7)	0.14(-0.39, 0.68)	-0.02(-1.21, 1.17)	0.26(-0.93, 1.46)	0.75(-0.45, 1.94)	-0.35(-1.55, 0.84)	0.06(-1.14, 1.25)
zBMI (= Obese) x survey year (= 2014) x sex (= females)) x school grade(=Grade 7)	0.14(-0.81, 1.09)	-0.64(-2.81, 1.52)	1.02(-1.07, 3.11)	1.32(-0.79, 3.42)	0.72(-1.43, 2.88)	-1.65(-3.74, 0.44)
zBMI (= Thinness) x survey year (= 2018) x sex (= females)) x school grade(=Grade 7)	-0.29(-1.03, 0.45)	-1.06(-2.72, 0.59)	-0.13(-1.77, 1.52)	-0.29(-1.95, 1.37)	0.62(-1.04, 2.28)	-0.43(-2.08, 1.22)
zBMI (= Underweight) x survey year (= 2018) x sex (= females)) x school grade(=Grade 7)	-0.14(-0.66, 0.38)	0.07(-1.10, 1.24)	-0.32(-1.49, 0.84)	1.46(0.29, 2.63)	-1.55(-2.72, -0.37)	-0.54(-1.71, 0.63)
zBMI (= Overweight) x survey year (= 2018) x sex (= females)) x school grade(=Grade 7)	0.13(-0.39, 0.65)	0.34(-0.82, 1.50)	1.08(-0.08, 2.24)	-0.19(-1.35, 0.98)	-0.47(-1.63, 0.68)	-0.11(-1.26, 1.05)
zBMI (= Obese) x survey year (= 2018) x sex (= females)) x school grade(=Grade 7)	0.39(-0.52, 1.31)	-0.69(-2.77, 1.40)	2.02(-0.03, 4.06)	0.74(-1.28, 2.76)	1.27(-0.79, 3.33)	-1.46(-3.50, 0.58)
zBMI (= Thinness) x survey year (= 2006) x sex (= females)) x school grade(=Grade 9)	-0.22(-1.12, 0.69)	-0.12(-2.15, 1.92)	-1.24(-3.24, 0.76)	-0.98(-3.03, 1.07)	1.36(-0.66, 3.38)	-0.27(-2.31, 1.78)
zBMI (= Underweight) x survey year (= 2006) x sex (= females)) x school grade(=Grade 9)	-0.26(-0.82, 0.29)	-0.55(-1.80, 0.70)	0.23(-1.02, 1.47)	0.13(-1.11, 1.38)	-0.02(-1.26, 1.22)	-1.24(-2.49, 0.01)
zBMI (= Overweight) x survey year (= 2006) x sex (= females)) x school grade(=Grade 9)	-0.34(-0.92, 0.24)	-0.27(-1.56, 1.03)	-0.28(-1.57, 1.01)	-0.55(-1.85, 0.76)	-0.11(-1.41, 1.18)	-0.47(-1.78, 0.84)
zBMI (= Obese) x survey year (= 2006) x sex (= females)) x school grade(=Grade 9)	-0.32(-1.39, 0.74)	-0.56(-2.95, 1.83)	-1.42(-3.79, 0.94)	1.24(-1.15, 3.62)	0.15(-2.25, 2.55)	-0.92(-3.31, 1.46)

© 2024 Chen S et al. *JAMA Psychiatry.*

zBMI (= Thinness) x survey year (= 2010) x sex (= females)) x school grade(=Grade 9)	-0.54(-1.45, 0.37)	0.46(-1.57, 2.49)	-1.32(-3.34, 0.70)	0.10(-1.93, 2.14)	-0.18(-2.26, 1.90)	-1.90(-3.94, 0.13)
zBMI (= Underweight) x survey year (= 2010) x sex (= females)) x school grade(=Grade 9)	-0.64(-1.20, -0.08)	-1.28(-2.53, -0.02)	-0.01(-1.26, 1.24)	-0.05(-1.30, 1.20)	-0.79(-2.03, 0.45)	-1.27(-2.52, -0.03)
zBMI (= Overweight) x survey year (= 2010) x sex (= females)) x school grade(=Grade 9)	0.02(-0.55, 0.59)	0.35(-0.92, 1.62)	-0.12(-1.39, 1.16)	0.49(-0.79, 1.77)	-0.29(-1.56, 0.98)	-0.34(-1.62, 0.95)
zBMI (= Obese) x survey year (= 2010) x sex (= females)) x school grade(=Grade 9)	-1.07(-2.09, -0.04)	-2.33(-4.63, -0.03)	-0.77(-3.04, 1.50)	-0.29(-2.58, 2.01)	-2.71(-5.04, -0.39)	-0.08(-2.36, 2.20)
zBMI (= Thinness) x survey year (= 2014) x sex (= females)) x school grade(=Grade 9)	-0.67(-1.56, 0.22)	-1.00(-2.97, 0.97)	-1.57(-3.55, 0.41)	0.94(-1.05, 2.93)	-0.53(-2.55, 1.48)	-1.35(-3.36, 0.66)
zBMI (= Underweight) x survey year (= 2014) x sex (= females)) x school grade(=Grade 9)	-0.31(-0.86, 0.25)	-0.23(-1.48, 1.02)	0.26(-0.98, 1.51)	-0.35(-1.60, 0.90)	-0.34(-1.57, 0.90)	-0.95(-2.19, 0.29)
zBMI (= Overweight) x survey year (= 2014) x sex (= females)) x school grade(=Grade 9)	0.12(-0.45, 0.69)	0.68(-0.59, 1.96)	-0.10(-1.37, 1.16)	0.21(-1.07, 1.49)	-0.34(-1.60, 0.92)	0.20(-1.08, 1.48)
zBMI (= Obese) x survey year (= 2014) x sex (= females)) x school grade(=Grade 9)	0.30(-0.73, 1.32)	-0.01(-2.32, 2.29)	-1.95(-4.24, 0.34)	3.62(1.35, 5.89)	1.24(-1.10, 3.57)	-1.24(-3.49, 1.01)
zBMI (= Thinness) x survey year (= 2018) x sex (= females)) x school grade(=Grade 9)	-0.34(-1.20, 0.51)	0.07(-1.83, 1.98)	-0.25(-2.13, 1.63)	-0.72(-2.66, 1.22)	0.30(-1.62, 2.21)	-1.37(-3.28, 0.54)
zBMI (= Underweight) x survey year (= 2018) x sex (= females)) x school grade(=Grade 9)	-0.11(-0.65, 0.43)	-0.53(-1.75, 0.69)	0.05(-1.15, 1.26)	1.38(0.16, 2.59)	-0.30(-1.50, 0.91)	-1.22(-2.42, -0.01)
zBMI (= Overweight) x survey year (= 2018) x sex (= females)) x school grade(=Grade 9)	-0.11(-0.67, 0.44)	0.32(-0.92, 1.56)	-0.14(-1.38, 1.10)	-0.16(-1.41, 1.09)	-0.09(-1.32, 1.14)	-0.44(-1.69, 0.81)
zBMI (= Obese) x survey year (= 2018) x sex (= females)) x school grade(=Grade 9)	-0.26(-1.25, 0.73)	-0.79(-3.01, 1.42)	-0.49(-2.69, 1.71)	1.99(-0.23, 4.21)	0.08(-2.15, 2.31)	-1.70(-3.90, 0.51)

© 2024 Chen S et al. *JAMA Psychiatry.*

eFigure 1. Generalised additive models of psychosomatic concerns as a function of zBMI, by survey year by sex by grade. Grey area presents 95%CIs of fitting on the function of zBMI. P values were extracted from the multi-level generalized additive model, with psychosomatic concerns as the outcome and smooth term of zBMI as the exposure, controlling for survey year, living with parents, sibling presence, academic pressure, been bullied, and smooth term of family affluence scale, screen time, and physical activity, as well as the random intercept and random slope for zBMI at the level of classroom, school, and country.

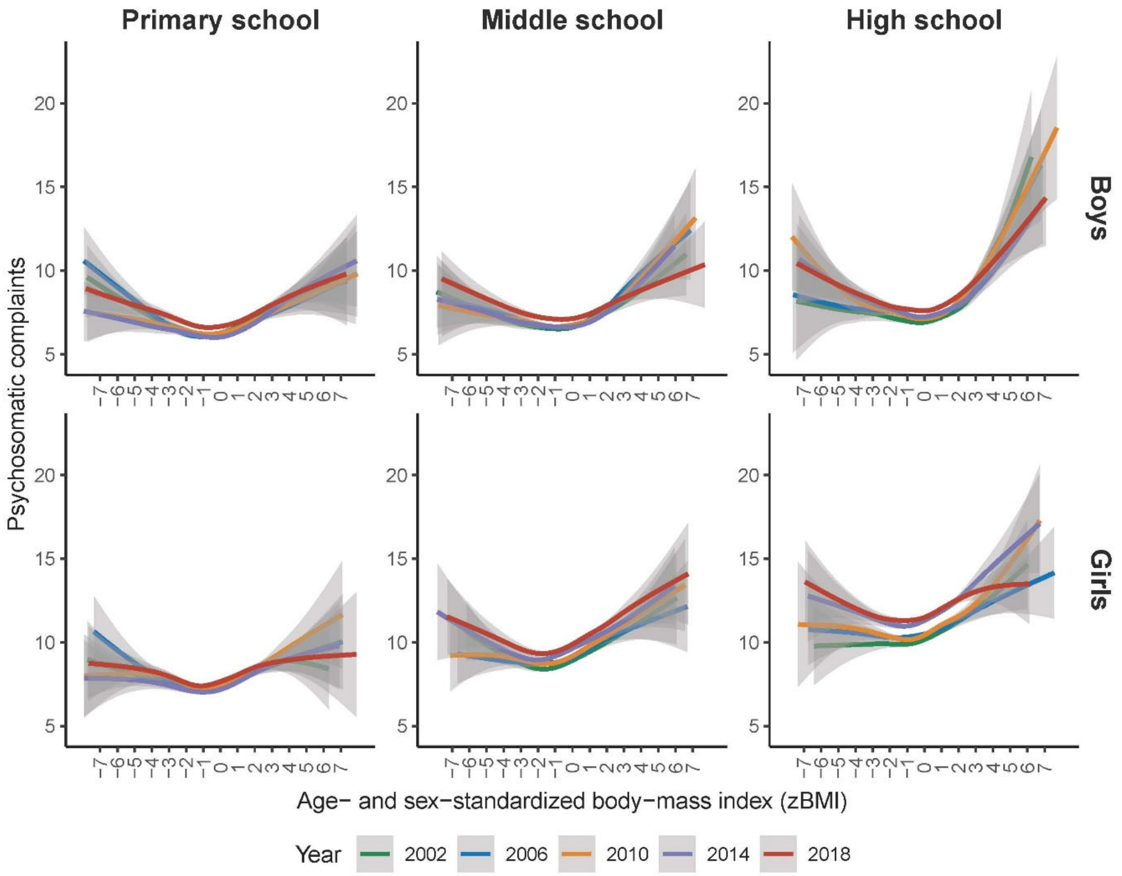

† For all panels, all P values for the spline of zBMI are <0.0001.

© 2024 Chen S et al. *JAMA Psychiatry.*

eFigure 2. Joint associations and their temporal trends of zBMI and sub-group variables with psychosomatic concerns. Points present the coefficients and the vertical lines present the 95% confidence interval (CI). Panel A visualizes the dose-dependent associations between zBMI (ref = normal weight) and psychosomatic concerns, which extracted from model 1.11 in table 2; Panel B visualizes the temporal trend on the association between zBMI and psychosomatic concerns, which extracted from model 1.11 in table 2 + zBMI categories (ref = normal weight) x survey year (ref = 2002); Panel C visualizes the effect modification of sex on the association between zBMI and psychosomatic concerns, which extracted from model 1.11 in table 2 + zBMI categories (ref = normal weight) x sex (ref = males); Panel D visualizes the effect modification of school grade on the association between zBMI and psychosomatic concerns, which extracted from model 1.11 in table 2 + zBMI categories (ref = normal weight) x school grade(ref = primary school); Panel E visualizes the effect modification of sex on the temporal trend of association between zBMI and psychosomatic concerns, which extracted from model 1.11 in table 2 + zBMI categories (ref = normal weight) x survey year (ref = 2002) x sex(ref = males); Panel F visualizes the effect modification of school grade on the temporal trend of association between zBMI and psychosomatic concerns, which extracted from model 1.11 in table 2 + zBMI categories (ref = normal weight) x survey year (ref = 2002) x school grade(ref = primary school); Panel G visualizes the joint effect modification of sex and school grade on the associations between zBMI and psychosomatic concerns, which extracted from model 1.11 in table 2 + zBMI categories (ref = normal weight) x sex (ref = males) x school grade(ref = primary school); Panel H visualizes the joint effect modification of sex and school grade on the temporal trend of associations between zBMI and psychosomatic concerns, which extracted from model 1.11 in table 2 + zBMI categories (ref = normal weight) x survey year (ref = 2002) x sex (ref = males) x school grade(ref = primary school).

© 2024 Chen S et al. *JAMA Psychiatry.*

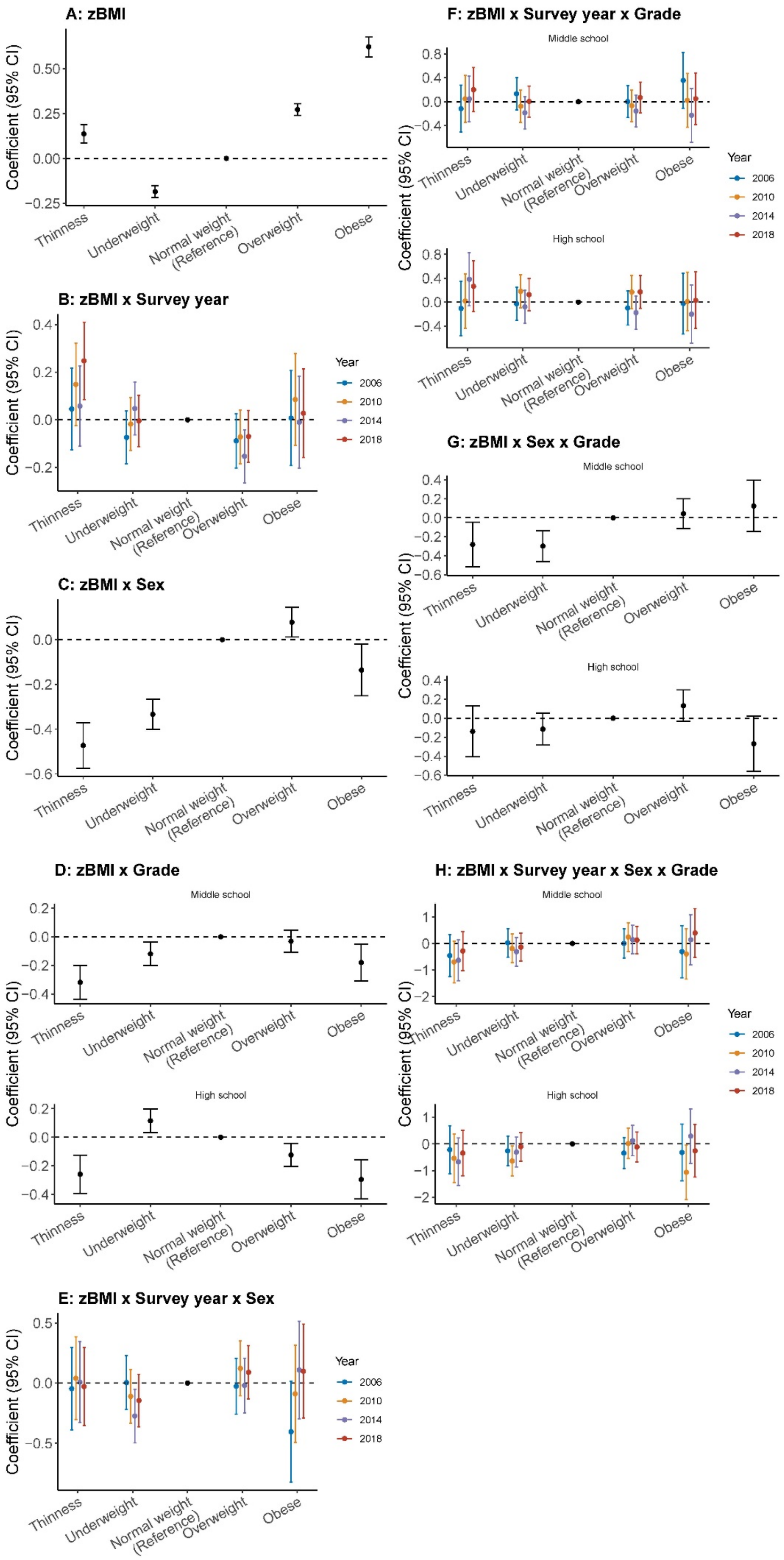

© 2024 Chen S et al. *JAMA Psychiatry.*

eFigure 3. Generalised additive models of psychosomatic concerns as a function of zBMI, by survey year, without imputation. Grey area presents 95%CIs of fitting on the function of zBMI. P values were extracted from the multi-level generalized additive model, with psychosomatic concerns as the outcome and smooth term of zBMI as the exposure, controlling for survey year, sex, grade, living with parents, sibling presence, academic pressure, been bullied, and smooth term of survey year, family affluence scale, screen time, and physical activity, as well as the random intercept and random slope for zBMI at the level of classroom, school, and country.

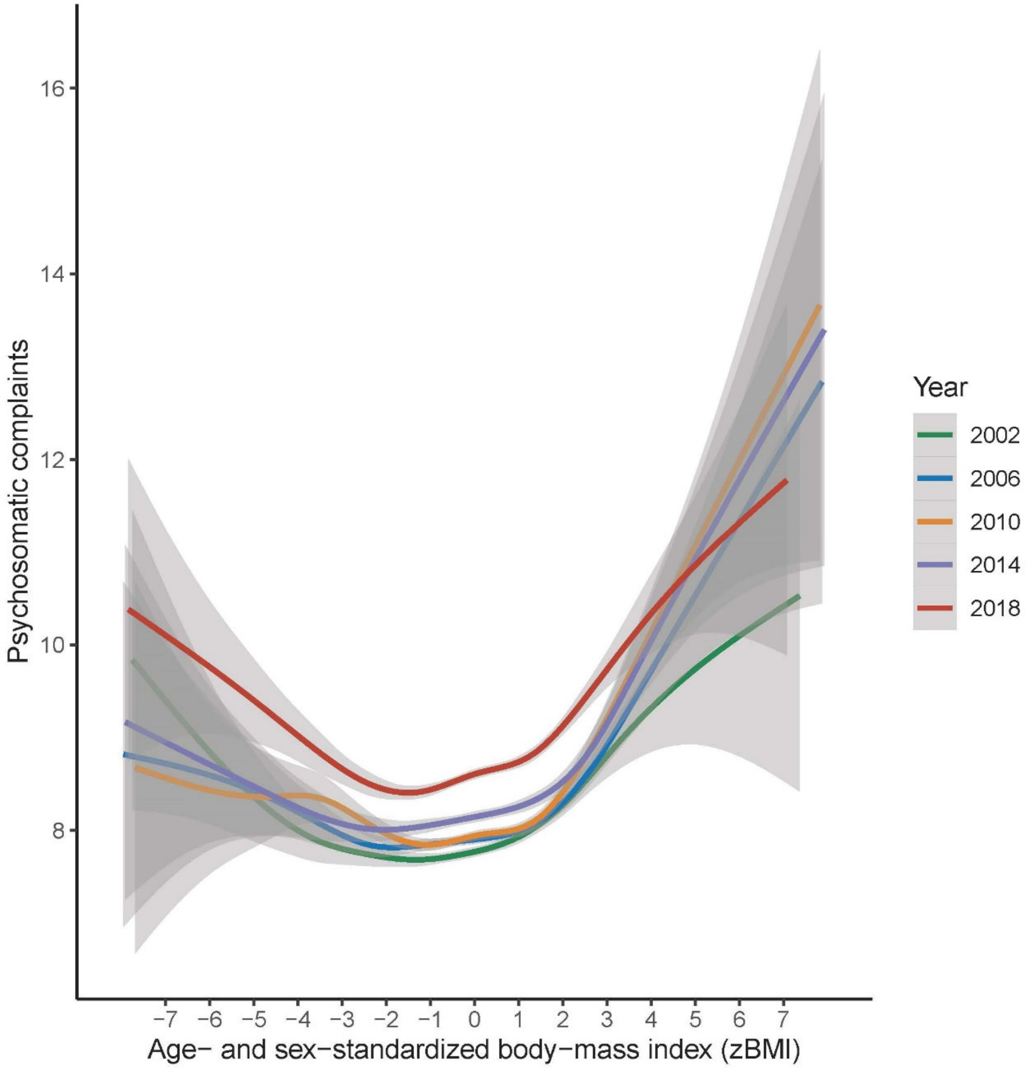

† P value for spline of zBMI < 0.0001

© 2024 Chen S et al. *JAMA Psychiatry.*

eFigure 4. Generalised additive models of psychosomatic concerns as a function of zBMI, by survey year by sex, without imputation. Grey area presents 95%CIs of fitting on the function of zBMI. P values were extracted from the multi-level generalized additive model, with psychosomatic concerns as the outcome and smooth term of zBMI as the exposure, controlling for survey year, grade, living with parents, sibling presence, academic pressure, been bullied, and smooth term of family affluence scale, screen time, and physical activity, as well as the random intercept and random slope for exposure at the level of classroom, school, and country.

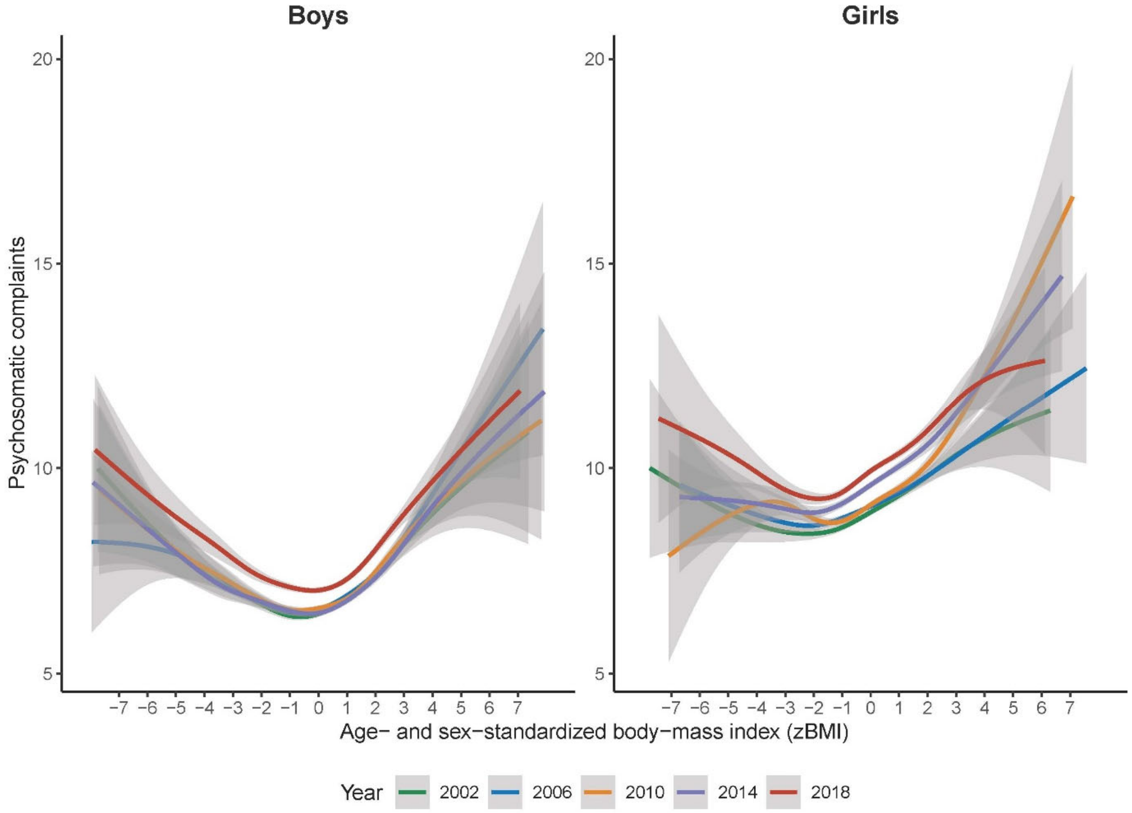

† For boys and girls, both P values for the spline of zBMI are <0.0001.

eFigure 5. Generalised additive models of psychosomatic concerns as a function of zBMI, by survey year by grade, without imputation. Grey area presents 95%CIs of fitting on the function of zBMI. P values were extracted from the multi-level generalized additive model, with psychosomatic concerns as the outcome and smooth term of zBMI as the exposure, controlling for survey year, sex, living with parents, sibling presence, academic pressure, been bullied, and smooth term of family affluence scale, screen time, and physical activity, as well as the random intercept and random slope for exposure at the level of classroom, school, and country.

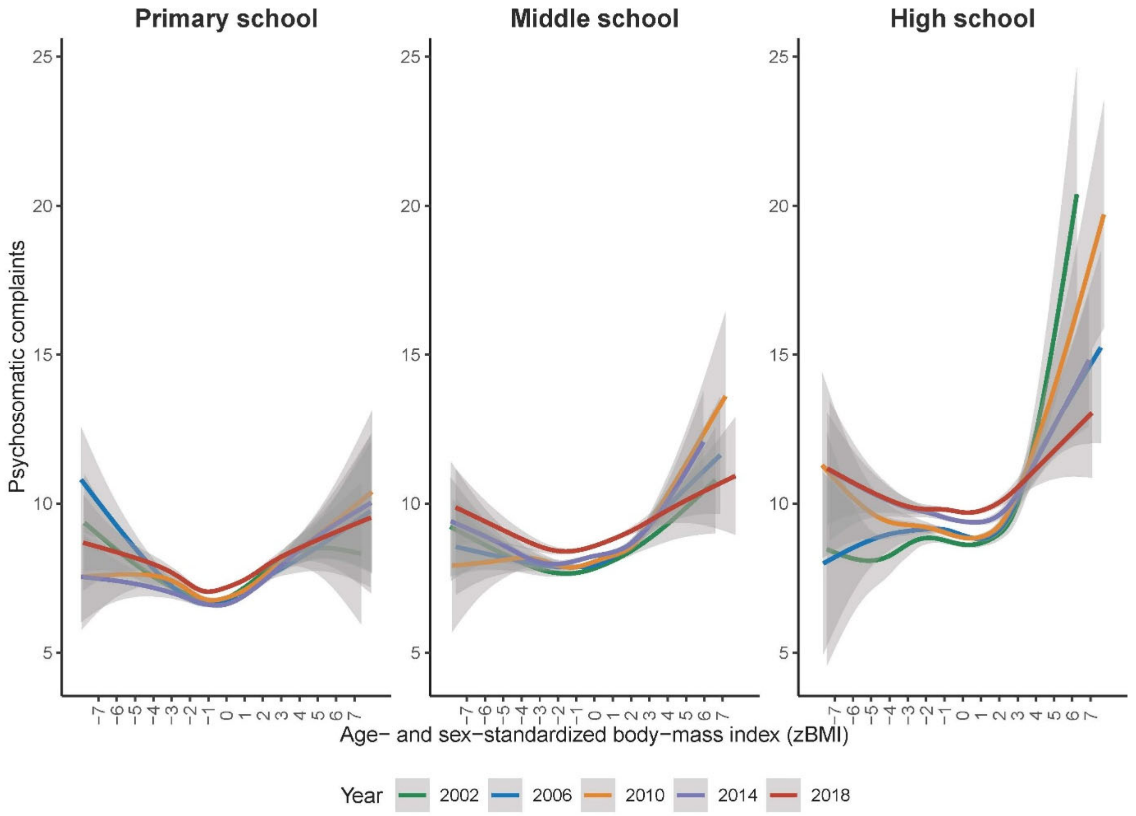

† For primary school, middle school, and high school, all P values for the spline of zBMI are <0.0001.

© 2024 Chen S et al. *JAMA Psychiatry.*

eFigure 6. Generalised additive models of psychosomatic concerns as a function of zBMI, by survey year by sex by grade, without imputation. Grey area presents 95%CIs of fitting on the function of zBMI. P values were extracted from the multi-level generalized additive model, with psychosomatic concerns as the outcome and smooth term of zBMI as the exposure, controlling for survey year, living with parents, sibling presence, academic pressure, been bullied, and smooth term of family affluence scale, screen time, and physical activity, as well as the random intercept and random slope for exposure at the level of classroom, school, and country.

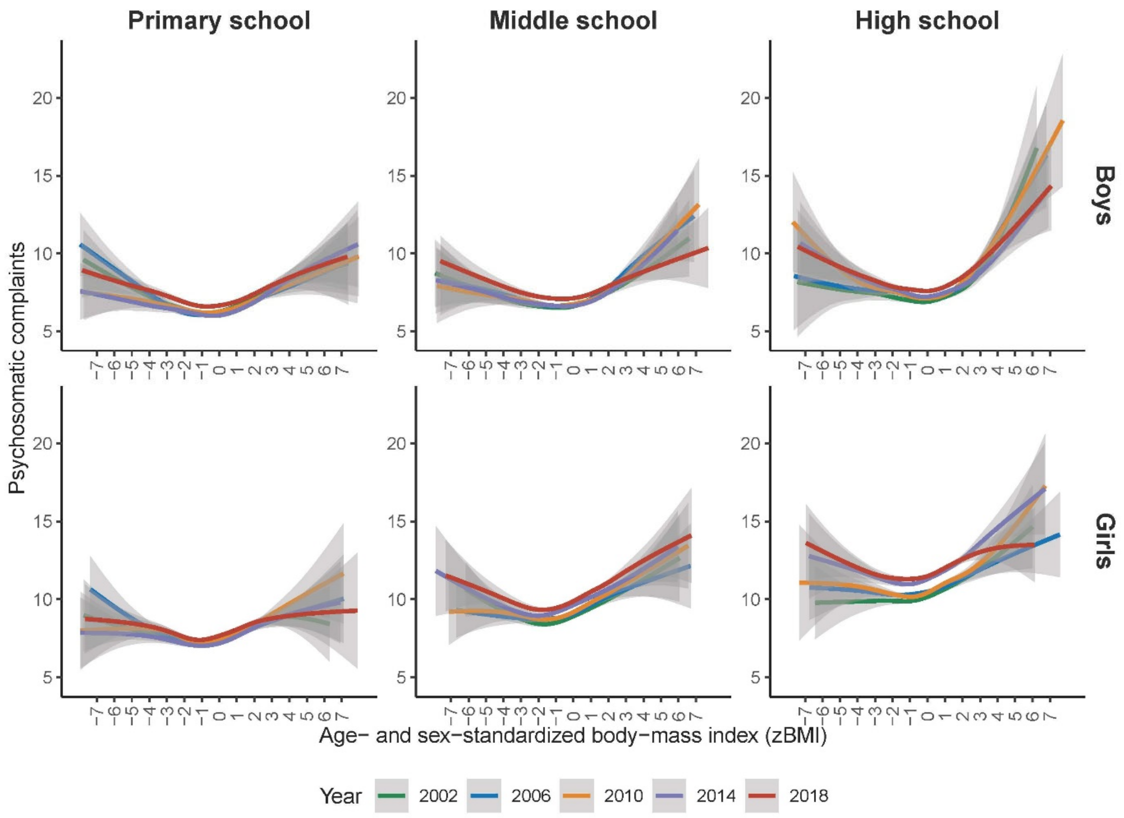

† For all panels, all P values for the spline of zBMI are <0.0001.

© 2024 Chen S et al. *JAMA Psychiatry.*

eFigure 7. Joint associations and their temporal trends of zBMI and sub-group variables with psychosomatic concerns, without imputation. Points present the coefficients and the vertical lines present the 95% confidence interval (CI). Panel A visualizes the dose-dependent associations between zBMI (ref = normal weight) and psychosomatic concerns, which extracted from model 1.11 in eTable 2; Panel B visualizes the temporal trend on the association between zBMI and psychosomatic concerns, which extracted from model 1.11 in eTable 2 + zBMI categories (ref = normal weight) x survey year (ref = 2002); Panel C visualizes the effect modification of sex on the association between zBMI and psychosomatic concerns, which extracted from model 1.11 in eTable 2 + zBMI categories (ref = normal weight) x sex (ref = males); Panel D visualizes the effect modification of school grade on the association between zBMI and psychosomatic concerns, which extracted from model 1.11 in eTable 2 + zBMI categories (ref = normal weight) x school grade(ref = primary school); Panel E visualizes the effect modification of sex on the temporal trend of association between zBMI and psychosomatic concerns, which extracted from model 1.11 in eTable 2 + zBMI categories (ref = normal weight) x survey year (ref = 2002) x sex(ref = males); Panel F visualizes the effect modification of school grade on the temporal trend of association between zBMI and psychosomatic concerns, which extracted from model 1.11 in eTable 2 + zBMI categories (ref = normal weight) x survey year (ref = 2002) x school grade(ref = primary school); Panel G visualizes the joint effect modification of sex and school grade on the associations between zBMI and psychosomatic concerns, which extracted from model 1.11 in eTable 2 + zBMI categories (ref = normal weight) x sex (ref = males) x school grade(ref = primary school); Panel H visualizes the joint effect modification of sex and school grade on the temporal trend of associations between zBMI and psychosomatic concerns, which extracted from model 1.11 in eTable 2 + zBMI categories (ref = normal weight) x survey year (ref = 2002) x sex (ref = males) x school grade(ref = primary school).

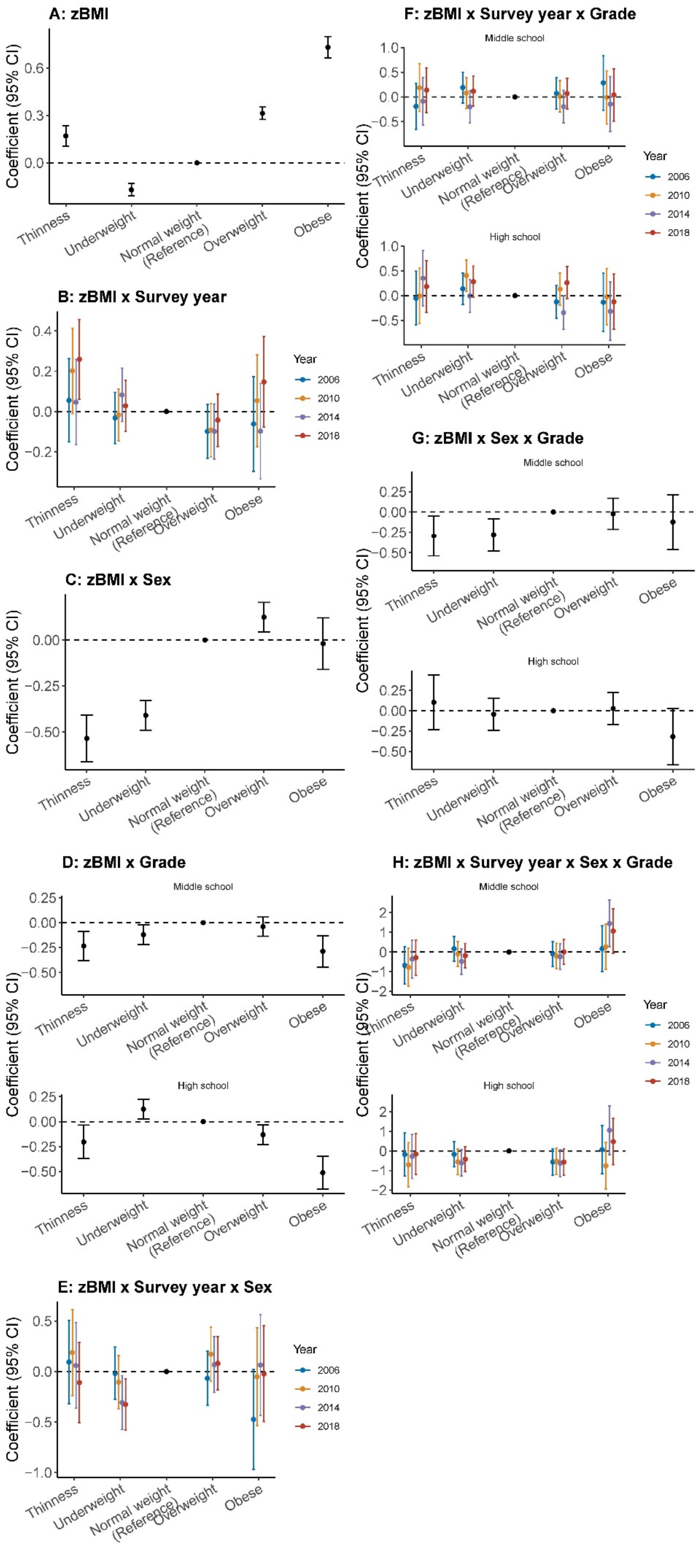

© 2024 Chen S et al. *JAMA Psychiatry.*

© 2024 Chen S et al. *JAMA Psychiatry.*
