# Supplementary material for: Dose-Dependent Association Between Body Mass Index and Mental Health and Changes Over Time
Source: JAMA Psychiatry. 2024 May 15;81(8):797–806. doi: 10.1001/jamapsychiatry.2024.0921 (PMC11097104; doi:10.1001/jamapsychiatry.2024.0921)
Supplement: Supplement 2. — Data sharing statement [file jamapsychiatry-e240921-s002.pdf]

Data Sharing Statement

Chen. Dose-Dependent Association Between Body Mass Index and Mental Health and Changes Over Time. *JAMA Psychiatry*. Published May 15, 2024.
doi:10.1001/jamapsychiatry.2024.0921

Data
Data available: No

Additional Information
Explanation for why data not available: The data are publicly available and can be accessed here (https://hbsc.org/data/).
